# MDAM-DRNet: Dual Channel Residual Network With Multi-Directional Attention Mechanism in Strawberry Leaf Diseases Detection

**DOI:** 10.3389/fpls.2022.869524

**Published:** 2022-07-08

**Authors:** Tingjing Liao, Ruoli Yang, Peirui Zhao, Wenhua Zhou, Mingfang He, Liujun Li

**Affiliations:** ^1^College of Computer and Information Engineering, Central South University of Forestry and Technology, Changsha, China; ^2^College of Food Science and Engineering, Central South University of Forestry and Technology, Changsha, China; ^3^Department of Civil, Missouri University of Science and Technology, University of Missouri-Rolla, Rolla, MO, United States

**Keywords:** detection of strawberry leaf diseases, color feature path, texture feature path, multidirectional attention mechanism, multidirectional attention mechanism dual channel residual network, ELU

## Abstract

The growth of strawberry plants is affected by a variety of strawberry leaf diseases. Yet, due to the complexity of these diseases' spots in terms of color and texture, their manual identification requires much time and energy. Developing a more efficient identification method could be imperative for improving the yield and quality of strawberry crops. To that end, here we proposed a detection framework for strawberry leaf diseases based on a dual-channel residual network with a multi-directional attention mechanism (MDAM-DRNet). (1) In order to fully extract the color features from images of diseased strawberry leaves, this paper constructed a color feature path at the front end of the network. The color feature information in the image was then extracted mainly through a color correlogram. (2) Likewise, to fully extract the texture features from images, a texture feature path at the front end of the network was built; it mainly extracts texture feature information by using an area compensation rotation invariant local binary pattern (ACRI-LBP). (3) To enhance the model's ability to extract detailed features, for the main frame, this paper proposed a multidirectional attention mechanism (MDAM). This MDAM can allocate weights in the horizontal, vertical, and diagonal directions, thereby reducing the loss of feature information. Finally, in order to solve the problems of gradient disappearance in the network, the ELU activation function was used in the main frame. Experiments were then carried out using a database we compiled. According to the results, the highest recognition accuracy by the network used in this paper for six types of strawberry leaf diseases and normal leaves is 95.79%, with an F1 score of 95.77%. This proves the introduced method is effective at detecting strawberry leaf diseases.

## Introduction

Strawberry is a sweet and sour delicious fruit prized by consumers that have high nutritional content and commercial value (Skrovankova et al., 2015). Strawberry has since become an important cash fruit crop in China (Lei et al., 2021). With the popularization of greenhouse cultivation technology, strawberries can be harvested year-round, and their cultivation area in China is expanding. However, high temperature and humidity in greenhouses offer favorable conditions for diseases and their outbreaks, leading to infections of strawberry plants that can seriously affect their yield of strawberry fruit (Wang et al., 2015). Because the symptomatic leaf spots of diseased strawberries show complex characteristics in both color and texture, their manual recognition method is time-consuming and laborious (Xiao et al., 2021) and it is thus more likely to miss the best time to intervene with control measures. Therefore, the development of a quick and reliable strawberry disease identification method could help fruit farmers implement timely control measures to reduce losses caused by disease, whose application value could be wide-ranging.

Color and texture are the two main visual attributes used to describe the disease spots that appear on infected plants. In traditional strawberry disease recognition, the types of disease spots are mainly determined manually, according to these two visual attributes. However, manual detection has several drawbacks, namely its slow speed, low accuracy, and large subjective error. In this respect, the field of plant science has advanced vigorously in recent years. Many researchers have proposed disease detection methods that rely instead on machine vision. For example, Kusumandari et al. (2019) proposed a strawberry leaf spot detection method based on color segmentation, for which the results showed a good detection effect. Yet, although this method can distinguish the diseased leaves from the background, this detection becomes impaired when the diseased spots are enlarged or the image quality is not sufficiently high. Robust strawberry disease image recognition inevitably requires fine-grained image classification, with more colors and irregular textures distinguishable. Therefore, during image processing, much color and texture information is apt to get lost, making accurate recognition more difficult. In addition, conventional image processing methods struggle to extract deeper feature information and often are not readily applicable to real environment settings. Recently, Huang et al. (2020) proposed PCNN-IPELM to detect peach diseases, and its detection effect is considered good. However, convolution only uses local information to calculate the target pixel, possibly leading to a loss of information given the lack of global features. Therefore, the key current problems in strawberry leaf disease identification are as follows: (1) the color and texture features of strawberry leaf disease spots are complex, and it is difficult to completely retain their crucial information. (2) It is hard to obtain deeper-level feature information using typical image processing methods, and their practical extension is weak. (3) In the process of image recognition, information loss can arise in the absence of global features.

To solve the problem (1), Kavitha and Suruliandi (2016) used GLCM and a color histogram to respectively extract the texture and color features of the image and then classified the image accordingly. Their experimental results demonstrated the classification effect is stronger when the texture feature is combined with RGB color space. However, GLCM entails abundant calculations, requiring much time. Fekriershad and Tajeripour (2017) had proposed using hybrid color local binary patterns (HCLBP), based on local binary patterns (LBP), to extract color and texture features, reducing the sensitivity of LBP to noise. They introduced an effective point selection algorithm to select the key points of the image and thus reduce the computational complexity; however, some color and texture features were abandoned when selecting such keys.

To solve the problem (2), and thereby extend the model's applicability to automated agricultural systems, Li and Chao (2021) proposed a semi-supervised small sample learning method to identify plant leaf diseases, which outperformed other related methods when less marker training data is available. While adding unlabeled data could improve the accuracy of that model in some cases, it may also render the model worse in other aspects. In the case of plant disease identification, marker data can also be used. Lv et al. (2020) designed DMS-Robust Alexnet, based on the backbone AlexNet structure. Combining extended convolution and multi-scale convolution, improved the feature extraction ability and showed strong robustness when applied to corn disease images collected in a natural setting. Although the extended convolution does increase the receptive field, not all inputs are involved in the calculation because of a gap in the convolution kernel. Zhang et al. (2020) proposed the FCM-NPGA algorithm to segment the image, to retain important texture information while removing noise points and edge points, finding it has high accuracy for detecting defects in apple fruit. But due to the partial loss of color and texture, that model is still limited for extracting key feature information.

To solve the problem (3), Wang et al. (2018) proposed a non-local module, to help the algorithm learn the relationship between different pixel positions, with promising results in the fields of action recognition, image classification, and target detection. This method, however, does not consider the relationship between different regional locations. Chen X. et al. (2020) introduced the channel attention mechanism into the dual-channel residual network and proposed B-ARNet, which can effectively improve the fine-grained classification effect. A drawback to this method is that multi-directional feature sequences are not well accounted for.

Accordingly, to tackle and simultaneously address all three primary problems, this paper proposes a new detection model for strawberry leaf diseases. Based on the ResNeXt network structure, this paper constructs a parallel color feature path and texture feature path at the front end of the network, which can retain the color and texture information in the original image more completely than traditional image processing methods. The two channels converge into a main frame road, to further extract the deep features. In this main road path, MDAM is introduced to improve the network's ability to extract critical features. The model can effectively detect strawberry leaf diseases and has high application value in agricultural automation systems. The main contributions and innovations of this paper are summarized as follows:

1. The color feature path was constructed by combining the color diagram and ResNeXt structure, enabling the effective extraction and description of the color feature map of a strawberry leaf disease image. It can obtain pivotal preliminary color feature information that then improves the disease detection ability of the network.
2. The texture feature path was constructed by combining ACRI-LBP and ResNeXt structure, to effectively extract and describe the texture feature map of a strawberry leaf disease image. Effective preliminary texture feature information is obtained, improving the ability of subsequent network detection.
3. This paper proposed a new attention mechanism—MDAM, used to obtain the weights of the feature layer in the road path of the main frame. The feature layer fused by color feature and texture feature path is inputted into MDAM, and the weights of different feature; information are obtained through a multi-directional comprehensive analysis. This method is helpful for extracting pertinent features, reducing the loss of main features from the strawberry leaf disease image, and improving the adaptability of the model to a complex environment. At the same time, the ELU activation function was used in MDAM-DRNet, adequately inhibiting the disappearance of the gradient.
4. The recognition rate of seven kinds of strawberry leaf images was 95.79%, and the F1 value was 95.77%. This indicates our model can accurately distinguish among strawberry leaf images displaying similar characteristics. Because of its robust classification performance in a complex natural environment, fruit farmers can use this method to judge whether strawberry leaves are infected with diseases, and to prevent and control strawberry diseases in advance, thereby ensuring the growth of strawberries and mitigating the economic losses caused by strawberry diseases.

## Related Work

**Figure 1:**
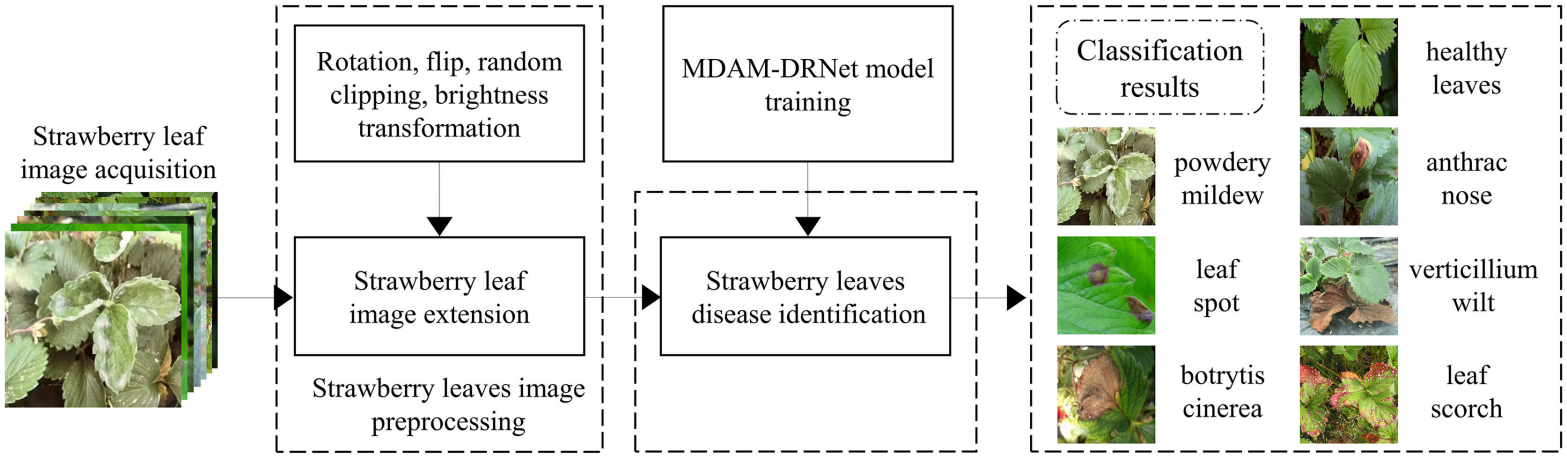
Schematic diagram of strawberry leaves disease identification.

In recent years, with the rapid development of machine vision technology, image processing techniques, and machine learning algorithms have been widely incorporated for the detection and classification of leaf diseases (Dhaka et al., 2021). Image processing techniques such as denoising and enhancement are the main methods applied to improve image quality. The use of appropriate image processing methods is conducive to improving recognition accuracy. Many researchers have made outstanding contributions in the field of image processing. Liu et al. (2021) proposed a self-attentional negative feedback network (SRAFBN) capable of achieving a real-time image super-resolution. This model can reconstruct the image texture more in line with human visual perception and has a better image enhancement effect. Chakraborty et al. (2021) proposed an apple leaf disease prediction method based on a multi-class support vector machine. To do this, first, the Otsu threshold algorithm and histogram equalization are used to segment the apple's infected disease area, and then a support vector machine identifies the disease type. Notably, the recognition accuracy achieved was high. To enable the accurate detection of plant diseases, researchers began to use the deep learning method to extract deep-seated features from images of diseased leaves. In this respect, Kundu et al. (2021) proposed a pearl millet disease prediction framework, based on the “internet of things” and interpretable machine learning, which can be used for accurate prediction of pearl millet outbreak and rust disease. Kim et al. (2021) proposed an improved vision-based strawberry disease detection method. Its PlantNet used in this method has a good ability to capture plant domain information. Xie et al. (2020b) proposed a real-time detector of grape leaf disease based on an improved deep-convolution neural network. The detection model Faster DR-IACNN achieved high accuracy when tested against a grape leaf disease data set. Finally, Yang et al. (2022) proposed a strawberry disease classification system that is based on deep learning; it provides a non-destructive, fast, and convenient classification scheme for diseases likely to occur in the process of strawberry planting. However, regarding the above plant disease detection methods, few studies have made full use of the color and texture features of disease spots that appear on leaves. In addition, the existing networks still face hurdles in fine-grained image recognition and their applied use in complex agricultural environments. Therefore, this paper proposes a new detection framework for strawberry leaf diseases that is based on a dual-channel residual network with a multi-directional attention mechanism (MDAM-DRNet). Our experimental results show that this method performs well in the fine-grained classification of strawberry leaf diseases, whose process is depicted in Figure 1.

## Materials and Methods

### Data Acquisition

To compile the data set used in experiments, online sources and orchard fields were used. The websites included Kaggle and social media, among others, yielding 2,753 photographs. Field images were collected from several strawberry picking gardens: Qingqing Strawberry Garden in Wangcheng District, Changsha City, Hunan Province; Zihui Farm Strawberry Picking Garden in Changsha Economic and Technological Development Zone; and the Shifang strawberry base at Hunan Agricultural University. The camera used was a Canon EOS R6, with an image pixel size of 2,400 × 1,600, and 3,841 strawberry disease images were taken. Because some websites lacked strict disease classification, some classification errors are inevitable. By consulting materials and asking experienced fruit farmers, we eliminated those images with poor quality and unclear objectives and reclassified the pictures having classification errors. Then, the above two data parts were integrated, for a total of 4,362 images, of which 1,106 were of early stages of strawberry leaf diseases. Because a large amount of data is needed for model training, the data was augmented by rotation, flip, random clipping, and brightness transformation tools. In this way, 17,440 images were finally obtained in a database. Table 1 lists the disease categories and corresponding data distribution of strawberry leaves used in this paper, including that for healthy strawberry, strawberry powdery mildew, strawberry leaf spot, strawberry *Botrytis cinerea*, strawberry anthracnose, strawberry verticillium wilt, and strawberry leaf scorch.

**Table 1:** Quantitative distribution of seven strawberry leaf images.

**Disease category**	**Example**	**Number**	**Proportion (%)**
*healthy leaves*	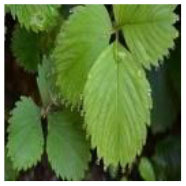	2,591	14.86
*powdery mildew*	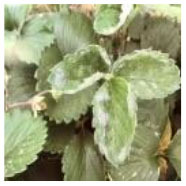	2,503	14.35
*leaf spot*	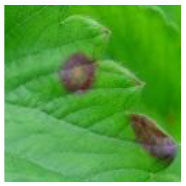	2,455	14.08
*Botrytis cinerea*	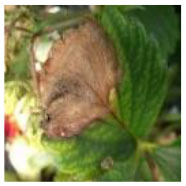	2,416	13.85
*anthracnose*	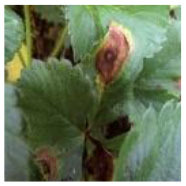	2,562	14.69
*verticillium wilt*	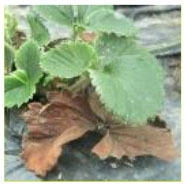	2,434	13.96
*leaf scorch*	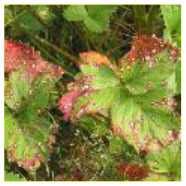	2,479	14.21

Combined with the six different disease images in Table 1, the leaf image characteristics of six strawberry diseases were analyzed. The above six diseases can differ starkly in the color and texture of their leaf spot symptoms. Their color characteristics are as follows: (1) Powdery mildew spots are white. (2) Leaf spot is purplish-red in the initial stage, gray in the center, turning purplish brown at the edge after expansion. (3) *Botrytis cinerea* spots appear yellowish-brown. (4) Anthracnose spots are reddish-brown or black in the early stage, brown in the center, and reddish-brown at the edge after expansion. (5) At the initial stage of verticillium wilt disease, its leaf spots are black-brown, but after expansion, they turn yellow-brown between leaf edges and leaf veins, with the new tender leaves appearing grayish-green or light brown. (6) The leaf scorch spot is purple to brown. The texture features of the leaf spots caused by different disease categories are as follows: (1) Powdery mildew is nearly round in the initial stage, whose edge is indistinct after radial expansion. (2) Leaf spot is a small round spot in the initial stage, taking the shape of a snake eye after expansion, and its wheel lines are fine and dense. In severe cases, the disease spots fuse together and the leaf dies. (3) *Botrytis cinerea* spots are large and “V”-shaped, and infected leaves die in severe cases. (4) Anthracnose spots are spindle-shaped with an uneven texture. (5) The initial stage of the verticillium wilt spot manifests a long strip shape in leaves; these wither in severe cases. (6) Leaf scorch leaves shrink, turn brown and inward, and wither with the severity. By comparing their respective color and texture, we can distinguish these six strawberry leaf diseases from healthy strawberry leaves. Therefore, this paper first extracts the texture and color features of strawberry leaves to obtain the shape feature information of a given disease. Next, in the subsequent identification of different strawberry leaf diseases by the neural network, the accuracy of strawberry leaf image classification can be significantly improved.

### Strawberry Leaf Disease Identification Based on the MDAM-DRNet Network

**Figure 2:**
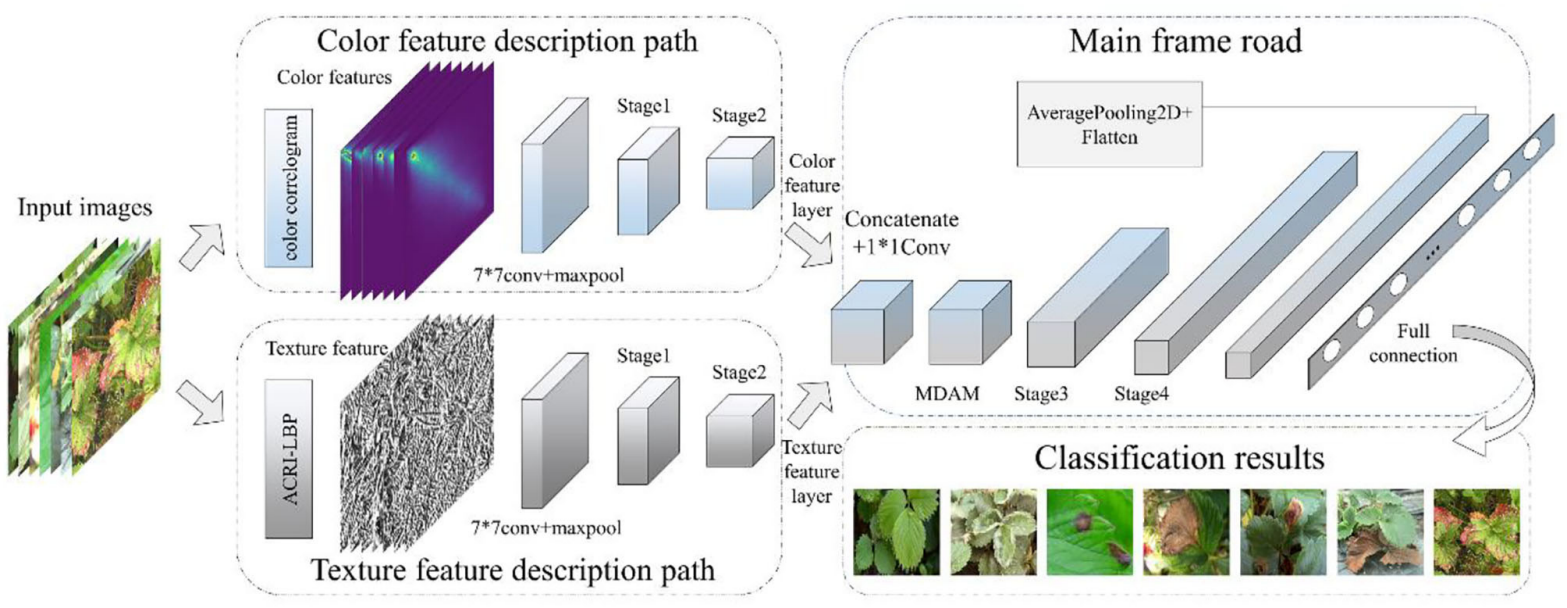
Structural diagram of MDAM-DRNet network.

As seen in Table 1, the diseases of strawberries are characterized by inconspicuous leaf spots small in area, hindering the manual diagnosis of the disease present and inevitably complicating disease identification, making it more likely to overshoot the best period to enact control measures. Therefore, the early monitoring of strawberry leaf diseases has more practical significance; more detailed features of this development stage ought to be extracted by a deep neural network. To solve the above problems, this paper proposes the MDAM-DRNet network, whose overall structure is illustrated in Figure 2. Firstly, the input data set passes through the color feature path and texture feature path in parallel. The color feature path includes the color correlogram and stage1 and stage2 of ResNeXt, through which the color feature layer can be extracted. The texture feature path includes ACRI-LBP and stage1 and stage2 of ResNeXt, through which the texture feature layer can be extracted. Then, the two-color feature and texture feature layers are merged into the main frame road path *via* concatenation, after which the MDAM attention mechanism is added to improve the recognition accuracy of a strawberry leaf disease image. The output of MDAM enters stage3 and stage4 of ResNeXt and continues to extract deep-seated feature information. Finally, after extracting the feature information from the network, the types of strawberry leaf disease mapped are categorized using the “softmax” classifier, whose classification results are outputted. The implementation process for the color feature path, texture feature path, and main frame road path is detailed below.

#### Color Feature Path

The color feature path is composed of the color correlogram and stage1 and stage2 of ResNeXt. Among them, the color correlogram is mainly used to extract and describe the color features in images of strawberry leaf diseases. Therefore, the following mainly introduces the implementation process of color feature extraction as well as describes the color correlogram.

The color correlogram is an expression of image color distribution. This feature not only describes the proportion of pixels of a certain color within the whole image but also reflects the spatial correlation between different color pairings (Jing et al., 1997). Research shows that a color correlation map has higher retrieval efficiency than does a color histogram or color aggregation vector (Wei-Ying and Hong Jiang, 1998). A color correlogram can express the proportion of pixels of a certain color in the whole image and the spatial correlation between pairs of a different color. Because the disease spots on strawberry leaves are small, the local correlation between colors is a more important consideration. Therefore, in order to reduce the space and time requirements, this paper sets the spatial distance D to a fixed value. The specific color extraction steps applied to a strawberry disease leaf image are shown in Table 2.

**Table 2:** Pseudo code of color correlation diagram.

**Algorithm 1 color correlogram**
**Input:** Color image *img*, Space distance *d*, Image length *L*, Image width *W*, Number of image channels *N* **Output:** Color correlogram cgram 1 **Begin** 2 **for** *x* **←** *0* **to** *L-1* **do** 3 **for** *y* **←** *0* **to** *W-1* **do** 4 **for** *t* **←** *0* **to** *N−1* **do** 5 /*Step 1: Take a point as the central pixel and obtain its pixel value*/ 6 *color_i ← Gets the pixel value of the (x, y, t) point* 7 /*Step 2: Obtain the eight field coordinates of the center point*/ 8 *neighbors ← Obtain the coordinates of 8 field points of (x, y, t)* 9 **for** *neighbors* **←***neighbors[0]* **to** *neighbors[7]* **do** 10 /*Step3: Gets the pixel value of a field*/ 11 *color_j ← Gets the pixel value of the i point* 12 /*Step 4: Record the number of color pairs (color_i, color_j)*/ 13 *cgram[color_i, color_j] ← cgram[color_i, color_j] + 1* 14 **end for** 15 **end for** 16 **end for** 17 **end for** 18 **return** *cgram* 19 **End**

**Figure 3:**
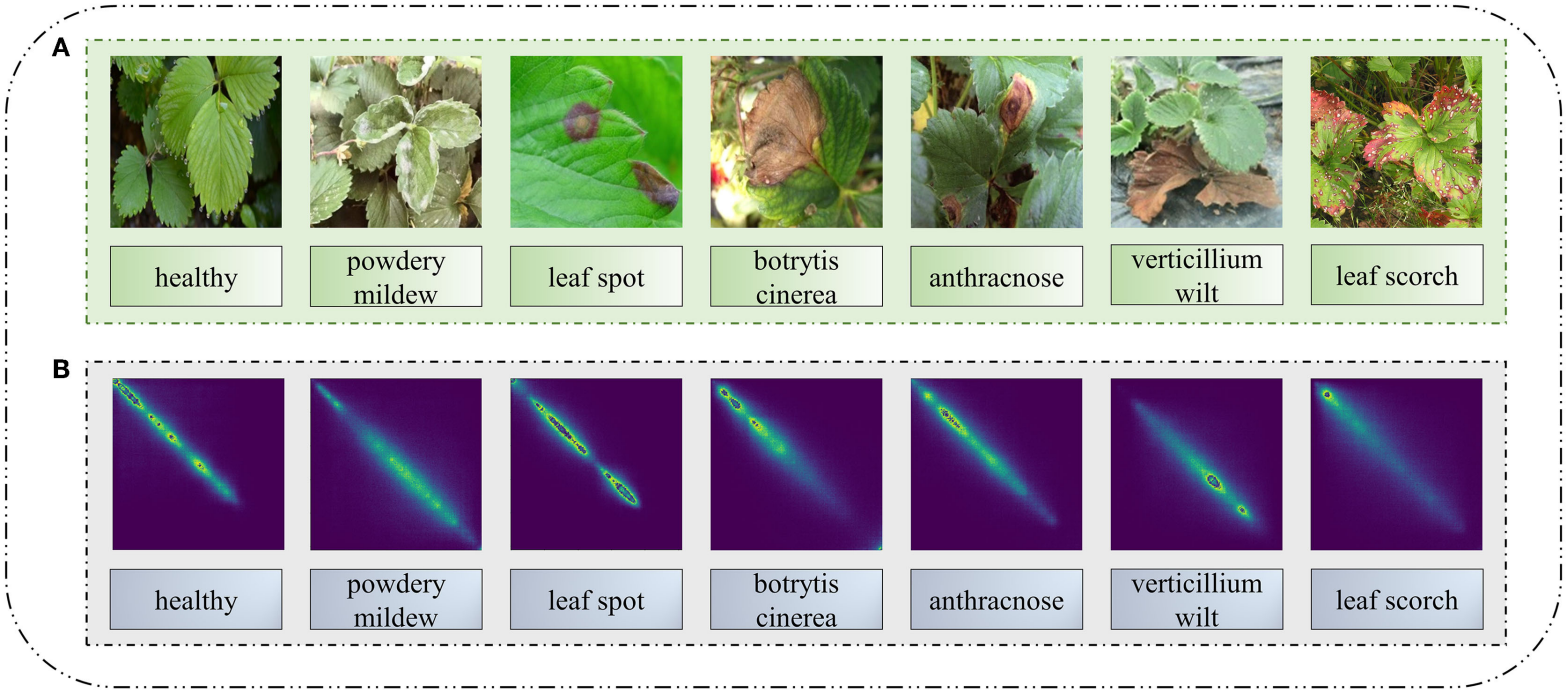
**(A,B)** Color feature map corresponding to five different strawberry leaf disease images.

A color correlogram can be understood as a table indexed by color pairs 〈*x, y*〉. Because the color correlogram only considers the local correlation between colors, this method is relatively simple and less computational taxing than extracting all color features of strawberry disease leaf images. Finally, the color features of different kinds of strawberry leaf disease images are extracted by the color correlogram (as seen in Figure 3). From the color features in that figure, they evidently differ considerably among the six diseases, indicating high discrimination.

#### Texture Feature Path

The texture feature pathway is composed of ACRI-LBP, in addition to stage1 and stage2 of ResNeXt. The ACRI-LBP algorithm is mainly used to describe and extract the texture features from images of strawberry leaf diseases. Therefore, the following focuses on the implementation process of texture feature description and extraction by ACRI-LBP.

LBP is a classical method for describing texture features (Tu et al., 2016). The original LBP operator is defined as taking the center pixel of the window as the threshold in a 3 × 3-window and comparing the gray value of eight adjacent pixels with it. If a surrounding pixel value is greater than the center pixel value, the position of that pixel is marked as 1; otherwise, it is 0. In this way, the eight points in the 3 × 3 neighborhood can be compared, to generate 8-bit binary numbers (usually converted into decimal numbers; i.e., LBP codes, for a total of 256); that is, the LBP value of the window's central pixel is derived, which may be used to reflect the texture information of the region. To resolve the issue arising when the LBP feature coding errs when the scale of the image changes, Guo et al. (2010) proposed the circular LBP (CLBP), which extends the 3 × 3 neighborhood to any neighborhood, by replacing the square neighborhood with a circular one, so as to obtain the LBP Operator with P sampling points in the circular region with a radius R. However, that LBP value will change once the image is rotated. Researchers have extended the LBP Operator to include rotation invariance (Mäenpää and Pietikäinen, 2005). Specifically, by continuously rotating the circular neighborhood the minimum LBP value is obtained, which then serves as the LBP feature of the central pixel. No matter how the image is rotated, the minimum eigenvalue in the field is finally found. For example, an initial LBP value in the circular neighborhood of 225, a series of LBP eigenvalues obtained after image rotation are 240, 120, 60, 30, 15, 135, and 195 respectively. In this group of LBP eigenvalues, if the smallest LBP eigenvalue is 15, the LBP characteristic for the central pixel of that circular neighborhood is 15. Given that the minimum value of the circular field corresponding to each pixel is different and fixed after rotation, the difference between pixels can also be clearly expressed by using the obtained minimum value. Therefore, RI-LBP (rotation invariant LBP) has rotation invariance and high description ability.

**Figure 4:**
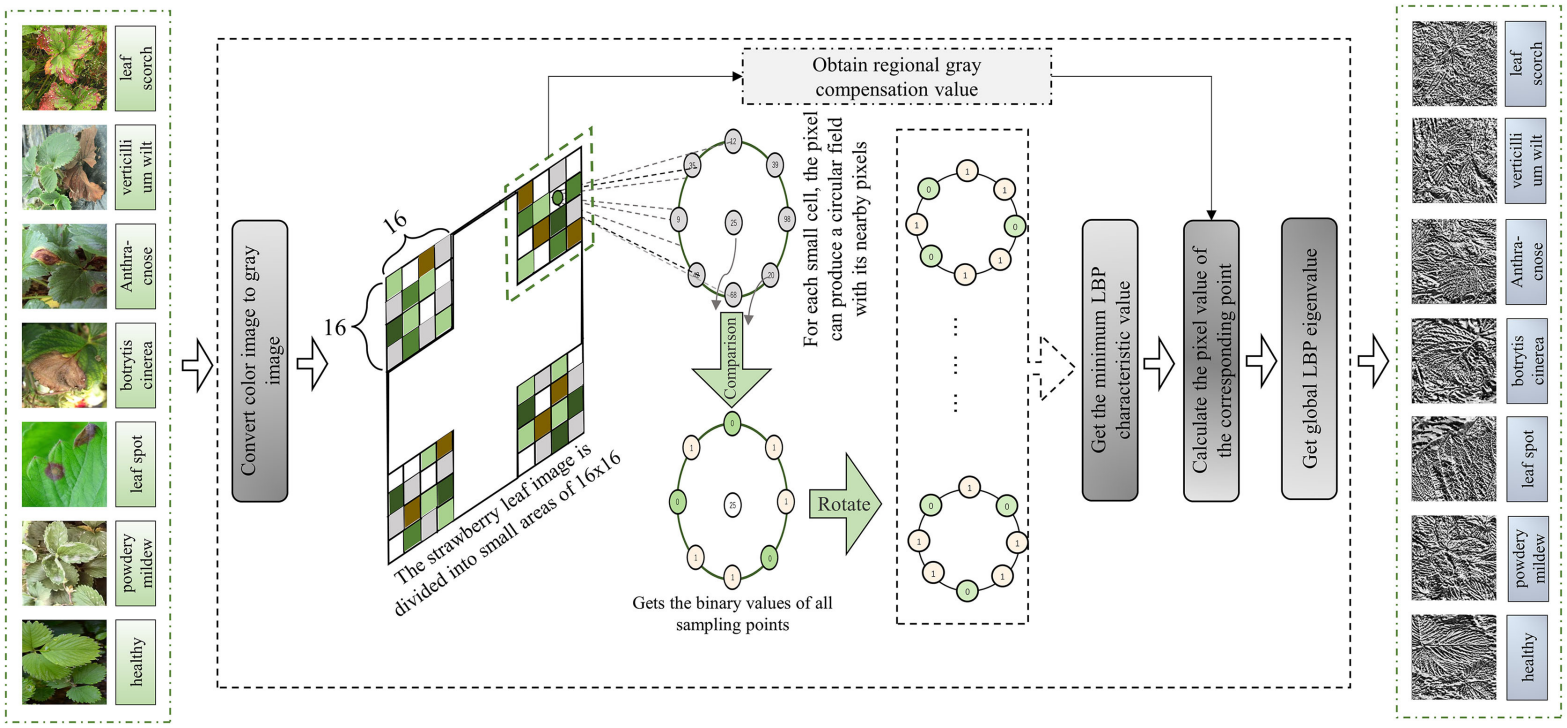
Schematic diagram of ACRI-LBP.

Now, considering that the LBP feature value obtained by RI-LBP is the minimum value obtained after the rotation of the circular field, the image may nonetheless be too dark because the feature value is too small, thus obscuring the texture features. Therefore, this paper adds area gray compensation to RI-LBP and proposes ACRI-LBP to extract the texture features. The schematic diagram of ACRI-LBP is presented in Figure 4. Specific steps for extracting texture features from a strawberry leaf image by ACRI-LBP are as follows:

(a) Firstly, the color image is transformed into a gray image.
(b) Divide the image into non-overlapping small areas, each 16 × 16 in size. Select the average value of *Agmax* and the maximum value of *Agmin* in the gray area of a pixel, and calculate the minimum value of *Agmax* in the gray area. The regional gray compensation value can then be calculated this way:

(1) $$\begin{array}{r} \text{Ac}={\frac{\text{Agmin}}{\text{Agmax}}}^{*}\text{Ag} \end{array}$$

(c) Select a pixel point in the region as the center point, whose coordinates are expressed as (*x*_*c*_, *y*_*c*_). Taking (*x*_*c*_, *y*_*c*_) as the center, draw a circle with radius *R*, select *P* sampling points with that circular area, and sampling points' coordinates as follows:

(2) $$\begin{array}{r} {\text{x}}_{\text{p}}={\text{x}}_{\text{c}}+\text{Rcos}\left(\frac{2\pi \text{p}}{\text{P}}\right) \end{array}$$

(3) $$\begin{array}{r} {\text{y}}_{\text{p}}={\text{y}}_{\text{c}}-\text{Rsin}\left(\frac{2\pi \text{p}}{\text{P}}\right) \end{array}$$

(d) If the coordinates of a sampling point are not at the center of the pixel, the bilinear interpolation method is used to obtain the coordinates of that sampling point. Set the coordinates of the four pixels around the sampling point as *Q*_11_ = (*x*_1_, *y*_1_), *Q*_12_ = (*x*_1_, *y*_2_), *Q*_21_ = (*x*_2_, *y*_1_), *Q*_22_ = (*x*_2_, *y*_2_), then derive its pixel value this way:

(4) $$\begin{array}{r} \text{f}\left(\text{x},\text{y}\right)=\left[{\text{x}}_{2}-\text{x x}-{\text{x}}_{1}\right]\left[\begin{array}{cc} \text{f}\left({\text{Q}}_{11}\right) & \text{f}\left({\text{Q}}_{12}\right)\\ \text{f}\left({\text{Q}}_{21}\right) & \text{f}\left({\text{Q}}_{22}\right) \end{array}\right]\left[\begin{array}{c} {\text{y}}_{2}-\text{y}\\ \text{y}-{\text{y}}_{1} \end{array}\right] \end{array}$$

(e) Next, compare the gray value of a point in the neighborhood with it. If the surrounding pixel value is greater than the central pixel value, then the position of that point is marked as 1; otherwise, it is marked as 0. In this way, the *P-*point in the neighborhood can generate a *P-*bit binary number after comparison; that is, the LBP value of the central pixel is obtained:

(5) $$\begin{array}{r} \begin{array}{c} \text{LBP}\left({\text{x}}_{\text{c},}{\text{y}}_{\text{c}}\right)=\sum_{\text{p}=0}^{\text{p}-1}2^{\text{p}}\text{s}\left({\text{i}}_{\text{p}},{\text{i}}_{\text{c}}\right) \end{array} \end{array}$$

(6) $$\begin{array}{r} \text{s}\left(\text{x}\right)=\{\begin{array}{c} 1\text{ if }{\text{i}}_{\text{p}}\geq {\text{i}}_{\text{c}}\\ 0\;\text{else} \end{array} \end{array}$$

where *i*_*p*_ denotes a pixel value of a neighborhood, and *i*_*p*_ denotes the center pixel value.

(f) Then, the binary values of the left turn bits are recycled, and then the decimal minimum value is taken as the eigenvalue of the current point.
(g) The eigenvalues of each pixel in each region are calculated. The pixel values of the corresponding points can be obtained by adding the eigenvalues of each pixel to the regional gray compensation value. Finally, the texture feature map is obtained by combining each pixel.

Because the radius is the amount actually selected according to the data set, and the smaller the radius, the finer the image texture, and the smaller the number of neighborhoods, the lower the brightness of the image.

#### The Main Frame Road Path

The main frame, road path entails the merged texture feature path and color feature path, the MDAM, and the stage3 and stage4 of ResNeX. The output feature layer of the color feature path and that of the texture feature path are fused after entering the main frame road path; hence, the fused output conveys the characteristics of color and texture. However, given the different contribution weights of these two features in the subsequent deep information extraction and disease classification performed by stage3 and stage4 of ResNeXt, we introduce MDAM to assign specific weights to different regions in the fused feature layer. Although stage1 and stage2 of the ResNeXt framework are both distributed in texture feature path and color feature path, stage3 and stage4 for extracting deep information and realizing classification functions are located in the main frame road path. Therefore, the frame of ResNeXt is introduced in the main frame road. To sum up, the following describes the implementation process of the main functions in the main frame road.

##### ResNeXt

When using a simple neural network for feature extraction, it is easy to lose the main features and thereby alter the classification effect. The deeper the network, the greater the possibility of decomposing the gradient. Residual network (ResNet) (He et al., 2016) can resolve this problem well. However, to improve model accuracy, the traditional ResNet needs to deepen the network. When deepening the network with more super parameters (such as the number of channels, filter size, etc.), the difficulty and computational overhead of network design will increase in tandem. Therefore, this paper uses the ResNeXt structure of Xie et al. (2017) as the basic framework for identifying strawberry leaf diseases. The introduction of ResNeXt not only can retain the residual structure of ResNet to preserve its excellent performance capabilities, but it also improves the recognition accuracy of strawberry leaves without exacerbating parameter complexity, by reducing and minimizing the number of super parameters needed and simplifying the network.

ResNeXt is based on ResNet, but the concept of cardinality is proposed on the structure of ResNet. Each layer of ResNet50 includes two modules: the identity block and the convolution block. The latter can change the network's dimensions but cannot be connected in series continuously, while the former is used to deepen the network and are connectable in a series. With the deepening of the network level, the things learned to become more complex, and more output channels arise. Therefore, while using identity blocks to deepen the network, it is also necessary to use convolution blocks to convert the dimensions, so that those features in the network's front can be transmitted to the feature layer in its back. Compared with previous networks, ResNeXt remains a popular network because of its few parameters, deep layers, and excellent classification ability and recognition effect.

##### MDAM

The attention mechanism (Luong et al., 2015; Cohn et al., 2016; Tu et al., 2016) originates from the simulation of the visual signal processing mechanism in humans. When people observe and recognize a target, they will focus on its prominent part and ignore some global and background information. This selective attention mechanism is consistent with the characteristics of the identification part in fine-grained image classification. Therefore, in order to extract the features of strawberry leaf images more thoroughly, a new attention mechanism—MDAM, is introduced here into ResNeXt. The MDAM is added after establishing the overall image feature connection layer. The MDAM model performs shallow mining on the overall image features *via* two-layer convolution. First, to each feature, MDAM assigns four weight coefficients (i.e., horizontal weight coefficient, vertical weight coefficient, left diagonal weight coefficient, and right diagonal weight coefficient). Because the attention mechanism in the Graph Attention Network (GAT) algorithm can assign different weights to adjacent nodes, it provides a way to extract deep-seated mutual features in adjacent directions (Velickovic et al., 2017). Therefore, we use the attention mechanism in the GAT algorithm to assign mutual weights to the four directional features. Next, each weighting coefficient is extended, by using the weight multiplication strategy and the maximum matching strategy, and the depth feature is generated through the convolution layer and an average pool. Meanwhile, the relationship between depth features is constructed using the MDAM algorithm (the weight feature map generated by the “softmax” function serves as the adjacency matrix of MDAM). By expanding the relationship between feature weight coefficients and structural features, MDAM can contribute to enhancing the overall classification of the whole strawberry leaf image. This should improve the final effect of fine-grained classification.

**Figure 5:**
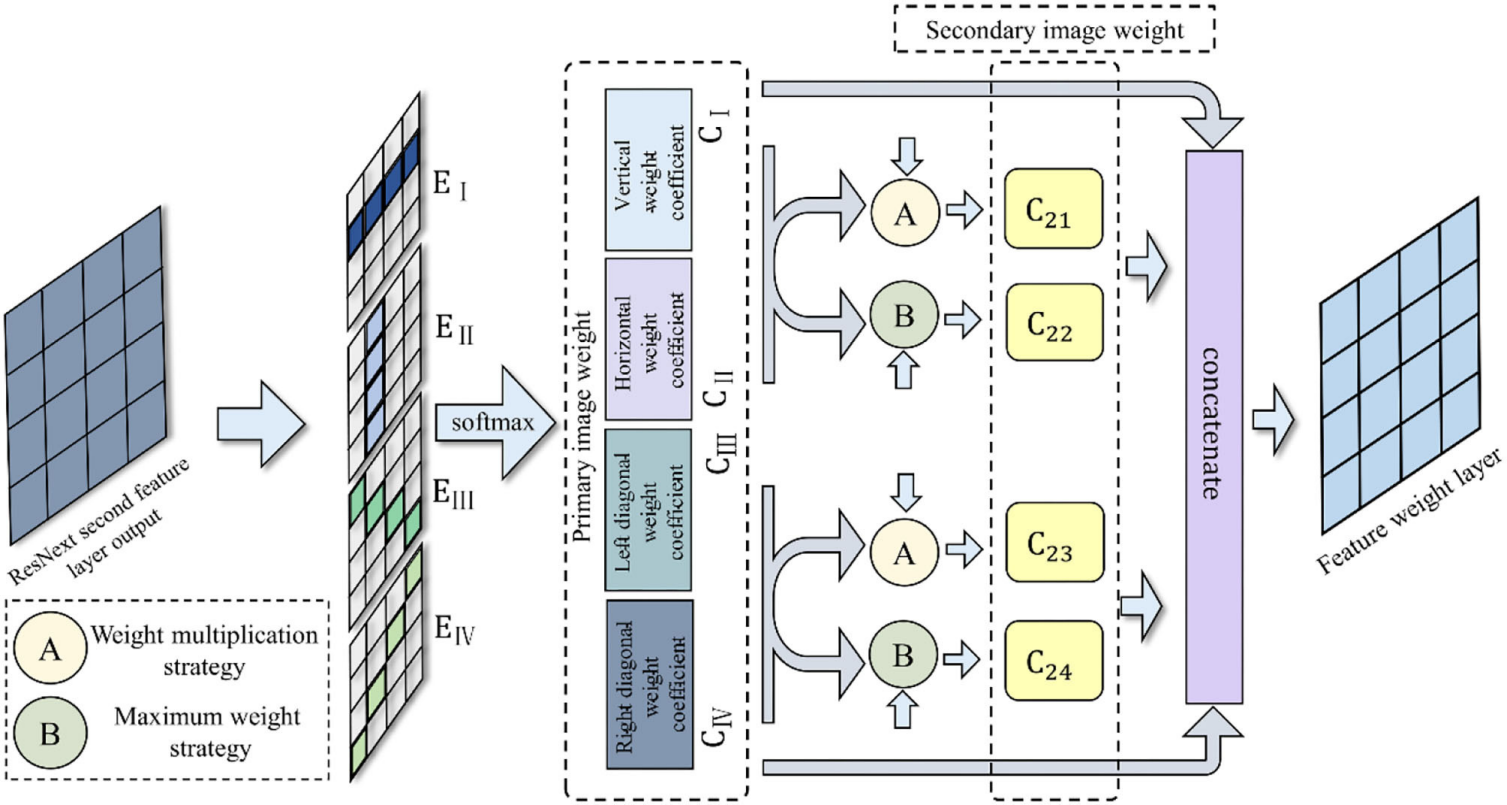
Algorithm block diagram of MDAM.

The MDAM proposed in this paper has three parts. Its algorithm block diagram appears in Figure 5.

Firstly, four primary image weight features *CCCC* are generated in the horizontal, vertical, left diagonal, and right diagonal directions. When a first-order image weight feature is generated, the feature vector set of the vertices of the first layer is $h={\left[h_{1},h_{2},...,h_{N}\right]}^{\text{T}}$, where *N* is the number of nodes in the graph (there are only four directions here, so *N* = 4). A weight matrix is needed to obtain the eigenvector of the next layer, so the weight matrix required is *W*. Then the feature vector set of the next layer can be obtained: $h^{{}^{\prime }}=\left[{h_{1}}^{{}^{\prime }},{h_{2}}^{{}^{\prime }},...,{h_{N}}^{{}^{\prime }}\right]$. For each node, the corresponding attention coefficient can be trained accordingly. The attention coefficient is thus given expressed as $e_{i,j}=a\left(W^{T}h_{i},W^{T}h_{j}\right)$. Next, the weight assigned by each vertex node *i* in each direction to node *j* on the feature sequence is obtained. Finally, the “softmax” function is implemented to regularize the attention coefficient, as shown in formula (7). The features extracted in multiple directions are highly complete, which is more conducive to extracting effective image features. Further, the weight distribution across multiple directions is more conducive to extracting the disease characteristics in different directions.

(7) $$\begin{array}{r} {\text{C}}_{\text{i}}=\sum_{\text{j}=1}^{\text{n}}\frac{\exp\left({\text{e}}_{\text{i},\text{j}}\right)}{\sum_{\text{k}=1}^{\text{n}}\exp\left({\text{e}}_{\text{i},\text{k}}\right)}{\text{h}}_{\text{j}} \end{array}$$

Secondly, the horizontal and vertical weight features are used to obtain the first secondary weight, *C*_21_, *via* the weight multiplication strategy given by Formula **(8)**. This weight multiplication strategy can mine the deep feature information and expand the weight coefficient by multiplying it with the minimum penalty. The weight multiplication can further amplify the influence of a weight coefficient—for example, the small weight may be 0.1^*^0.2, while the large weight may be 0.9^*^0.7–so as to obtain the extended feature. The horizontal and vertical weight features are then used to obtain the second secondary image weight, *C*_22_, by applying a maximum weight strategy. In the latter's Formula **(9)**, the maximum feature is deemed an effective feature and consistent with the minimum feature α multiple addition, where α is a decimal number between 0 and 1. This method takes the maximum as the main factor and considers another feature to obtain the comprehensive feature. The weight features of the left diagonal and right diagonal are multiplied to obtain the third secondary image weight, *C*_23_. Likewise, the weight features of the left diagonal and right diagonal are also multiplied to obtain the third secondary image weight *C*_24_. The specific formulae for obtaining *C*_21_, *C*_22_, *C*_23_, *C*_24_ weight are as follows:

(8) $$\begin{array}{r} {\text{C}}_{21}={\text{C}}^{*}\text{C}-\min\left(\text{C},\text{C}\right) \end{array}$$

(9) $$\begin{array}{r} {\text{C}}_{22}=\max\left(\text{C},\text{C}\right)+\alpha^{*}\min\left(\text{C},\text{C}\right) \end{array}$$

(10) $$\begin{array}{r} {\text{C}}_{23}={\text{C}}^{*},\text{C}-\min\left(\text{C},\text{C}\right) \end{array}$$

(11) $$\begin{array}{r} {\text{C}}_{24}=\max\left(\text{C},\text{C}\right)+\alpha^{*}\min\left(\text{C},\text{C}\right) \end{array}$$

Finally, the four types of weight features *C*_21_, *C*_22_, *C*_23_, *C*_24_ are matched to obtain the maximum value, which is used to supplement the results of four primary image weight coefficients *C, C, C, C*. In MDAM, these eight different image weight coefficients integrate the processed feature information in series through the concatenate function.

(12) $$\begin{array}{r} \text{MDAM}=\text{concatenate }\left(\left[\text{C},\text{C},\text{C},\text{C},{\text{C}}_{21},{\text{C}}_{22},{\text{C}}_{23}{,\text{C}}_{24}\right]\right) \end{array}$$

##### ELU Function

The output of upper nodes in ResNeXt and the input of lower nodes are connected by a ReLU activation function. Still, some neurons in ReLU may never get activated. Compared with ReLU, the ELU function does not have this “dead” problem, and it can effectively solve the problem of gradient disappearance (Clevert et al., 2015). Therefore, this paper selects the ELU activation function to replace ReLU. The ELU function is expressed this way:

(13) $$\begin{array}{r} \text{f}\left(\text{x}\right)=\{\begin{array}{c} \text{x if}\left(\text{x}>0\right)\\ \alpha \left({\text{e}}^{\text{x}}-1\right)\;\text{otherwise} \end{array} \end{array}$$

According to that formula, an output from ELU is maintained even if the input is negative. This ensures the advantages of the ReLU function are inherited while letting the ELU function solve the problem of gradient explosion in the network. Further, because the output mean of ELU is close to zero, its convergence speed is faster than that of the ReLU function. In addition, for the MDAM-DRNet network in this paper, without batch normalization, the ReLU network with > 30 layers will not converge, whereas incorporating the ELU function enables the network to reach high convergence despite more layers.

## Results

### Laboratory Environment

The experimental works were carried out on Windows 10 64-bit operating system equipped with a Core i9-9980xe CPU and NVIDA GeForce RTX 2080ti GPU. The software environment consisted of a CUDA Toolkit (v10.2), CUDNN (v7.6.5), Pycharm (v2019.3), Python (v3.7), and torch (v1.9.1), Numpy (v1.21.4), and OpenCV (v4.5.4.60). The experiments in this paper were all carried out in the same computing environment.

The unified input size of each image is 224^*^224. During the input process, the data set was expanded by horizontal flipping, small-angle rotation, and scaling, generating a total of 17440 images for analysis. This augmented data set was divided into a training set, testing set, and verification set in a ratio of 3:1:1. There are 10,464 images in the training set and 3,488 images in each test set and verification set.

Considering the hardware performance and training effect, the random gradient descent method was used to train the network. To do this, the size of each training and test was set to 24, that is, the batch size is 24, the epoch count is 200, and the momentum parameter is set to 0.9. The model used an Adam optimizer (Kingma and Ba, 2014), because the setting of a learning rate will affect the convergence speed and stability of the model. A callback function was added, the learning rate of the first 60 epochs was set to 0.0001, and the learning rate of the last 60 epochs reduced 10-fold; doing this increased the fitting speed and set the weight falloff to 0.0005.

### Evaluation Indicators

To evaluate the classification effect of the model, we selected accuracy, precision, recall, and F1 score as evaluation indicators. For a single category, the corresponding calculation formulae were as follows:

(14) $$\begin{array}{r} \text{Accuracy}=\frac{\text{TF}+\text{TP}}{\text{FP}+\text{TN}+\text{TP}+\text{FN}} \end{array}$$

(15) $$\begin{array}{r} \text{Precision}=\frac{\text{TP}}{\text{TP}+\text{FP}} \end{array}$$

(16) $$\begin{array}{r} \text{Recall}=\frac{\text{TP}}{\text{TP}+\text{FN}} \end{array}$$

(17) $$\begin{array}{r} \text{F1}=2^{*}\frac{{\text{Precision}}^{*}\text{Recall}}{\text{Precision}+\text{Recall}} \end{array}$$

where TP is the number of strawberry leaf disease samples predicted to be of class A that are actually class A (i.e., positive samples are tested as positive samples). FP is the number of strawberry leaf disease samples that are not predicted as class A but actually are of class A (negative samples are tested as positive samples). FN is the number of samples of strawberry leaf diseases predicted to be of class A yet is not actually class A (if no positive sample is detected, it is designated a positive sample). *Accuracy* corresponds to the proportion of samples correctly classified among all samples attempted; *Precision* is used to measure the number of correctly predicted samples whose predictions were positive; *Recall* is used to measure the number of correct predictions among the real positive samples; the *F*1 score is used to weigh precision and recall in the case of binary classification. For the case of multiple categories, the F1 score must synthesize the calculation results of the evaluation indicators of each category. Such a macro-F1 has the advantage of treating all categories equally (Opitz and Burst, 2019); hence, Macro-F1 was selected as the evaluation index, expressed by *F*1_*macro*_, whose calculation formula is as follows:

(18) $$\begin{array}{r} {\text{Precision}}_{\text{macro}}=\frac{\sum_{\text{i}=1}^{\text{n}}{\text{Precision}}_{\text{i}}}{\text{n}} \end{array}$$

(19) $$\begin{array}{r} {\text{Recall}}_{\text{macro}}=\frac{\sum_{\text{i}=1}^{\text{n}}{\text{Recall}}_{\text{i}}}{\text{n}} \end{array}$$

(20) $$\begin{array}{r} {\text{F1}}_{\text{macro}}=2^{*}\frac{{\text{Precision}}_{\text{macro}}^{*}{\text{Recall}}_{\text{macro}}}{{\text{Precision}}_{\text{macro}}+{\text{Recall}}_{\text{macro}}} \end{array}$$

where *i* represents class *i*, *Precision*_*macro*_ can be regarded as averaging the precision of *i* categories, and *Recall*_*macro*_ can be regarded as averaging the recall of *i* categories.

### Performance and Analysis

#### Comparative Experiment Between ACRI-LBP and Other Texture Extraction Methods

**Figure 6:**
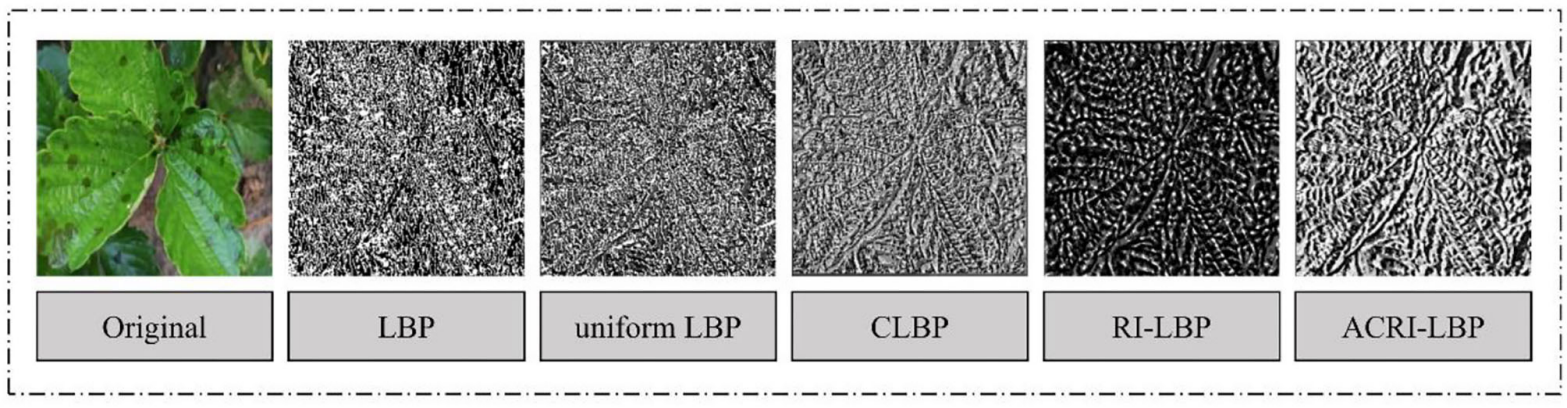
Texture feature images extracted by different methods.

In order to verify the performance of ACRI-LBP, four methods—LBP, CLBP, uniform LBP (Ojala et al., 2002), and RI-LBP—were compared with ACRI-LBP to test the effect of different methods for extracting texture features from the images (Figure 6). We can see that the texture features extracted by the CLBP, RI-LBP, and ACRI-LBP methods are relatively clear. To test the influence of different texture feature extraction methods on the network recognition rate, we replaced ACRI-LBP in MDAM-DRNet with LBP, CLBP, uniform LBP, or RI-LBP, and carried out experiments on the same self-made data set. The scores of accuracy, precision, recall, and F1 were the evaluation indicators.

According to these experimental results (Table 3), the accuracy attained by the RI-LBP method was 95.79%, and whose F1 score was 95.77%. Compared with that, our ACRI-LBP had an accuracy of 3.22% higher and an F1 score of 3.17% higher. Compared with the original LBP, accuracy increased by 10.04%, and F1 increased by 10.02% when using ACRI-LBP. This proved that ACRI-LBP is effective at improving the accuracy of network recognition and robust method to extract image texture features.

**Table 3:** Experimental results using different texture extraction methods.

**Radius**	**Accuracy**	**Precision_**macro**_**	**Recall_**macro**_**	**F1_**macro**_**
	**(%)**	**(%)**	**(%)**	**(%)**
LBP	85.75	85.76	85.73	85.75
Uniform LBP	87.90	87.93	87.91	87.92
CLBP	91.06	91.07	91.14	91.11
RI-LBP	92.57	92.59	92.61	92.60
ACRI-LBP	95.79	95.76	95.79	95.77

#### Verification Experiment for the Illumination Robustness of ACRI-LBP

**Figure 7:**
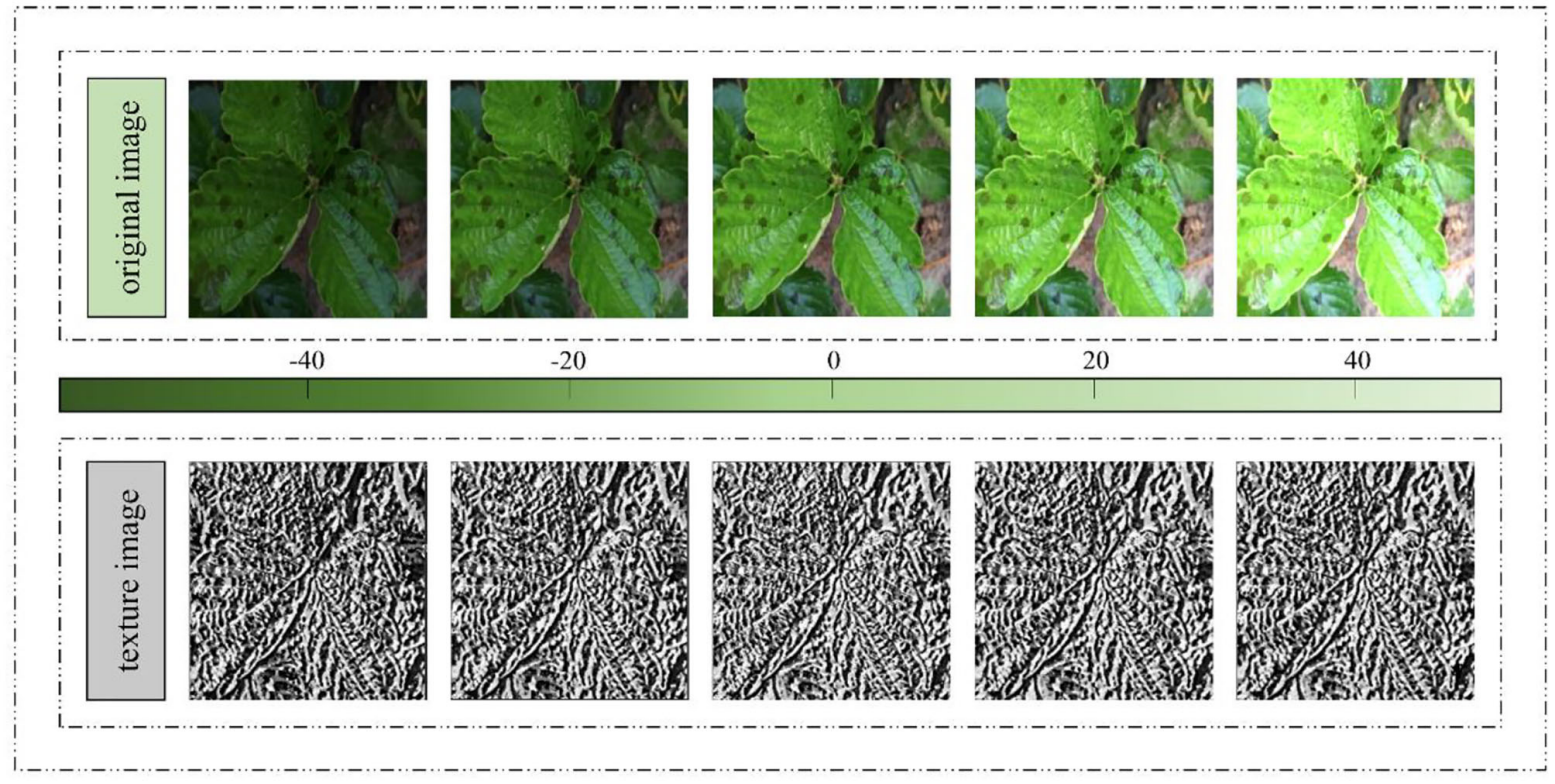
Texture feature map of the same image extracted under different brightness.

According to the above description of ACRI-LBP's properties, it only considers the size relationship between the center and adjacent pixel intensity, thus being invariant to a uniform change in whole-image intensity and robust to illumination changes. Therefore, different brightness processing was applied to the same image of a diseased strawberry leaf to obtain five pictures of increasing brightness, from left to right, as shown in Figure 7. Then, ACRI-LBP was used to extract their texture features, whose final texture features also appear in Figure 7.

In that figure, the texture features extracted by ACRI-LBP are consistent across images differing in brightness. This indicated that extraction is not affected by light, and that texture features are clearly extractable even for strawberry leaf images obtained under low light conditions. Therefore, ACRI-LBP's texture extraction can safeguard the utility of the image from low image quality caused by uneven illumination or low brightness. This proved that texture feature extraction *via* ACRI-LBP can effectively improve the subsequent recognition rate of various strawberry leaf disease images.

#### Effect of the ACRI-LBP Domain Radius on Image Recognition of Strawberry Leaf Diseases

**Figure 8:**
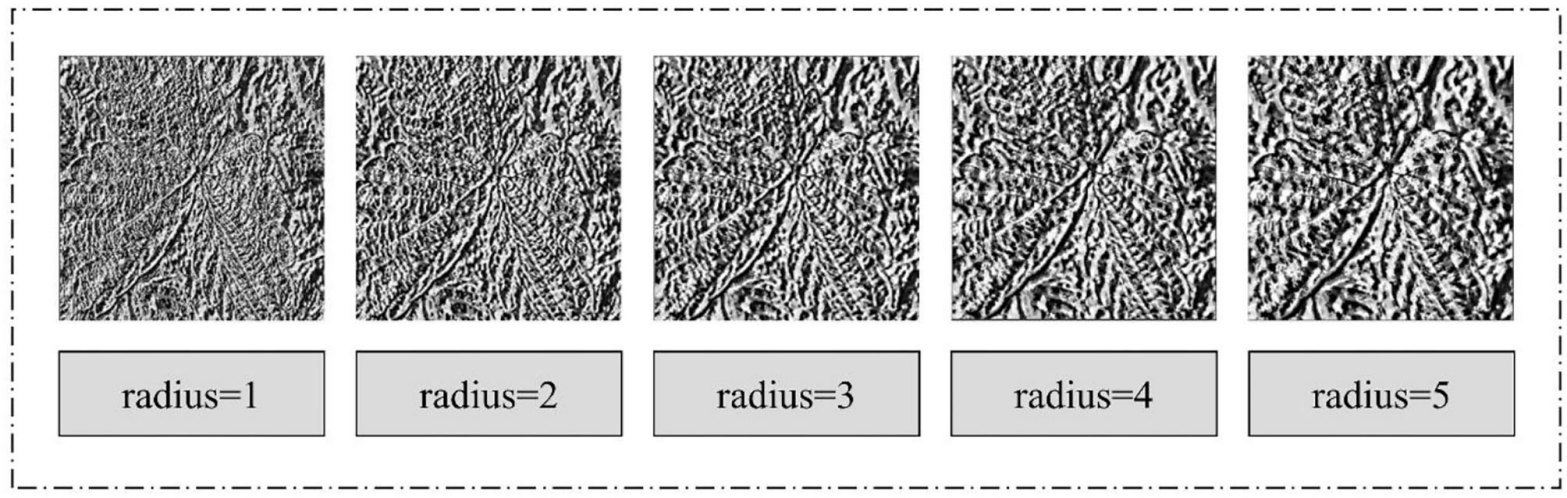
Texture feature map extracted from the same image after selecting different domain radius.

Changing the domain radius can generate different scale texture features. Accordingly, we selected four different radii (1, 2, 3, and 4) as the domain radius to extract the texture features of a strawberry leaf disease image. When different texture features are inputted into the MDAM-DRNet network, different strawberry leaf disease image recognition rates are obtained. Figure 8 shows the texture feature map extracted for the same image after applying the different field radii.

Evidently, the feature texture extracted is clearest when the domain radius is 3. The specific reason for this is that the regional feature information associated with other parts cannot be extracted in a domain radius that is too small; conversely, extracting more detailed location feature information is precluded when too large a domain radius is used. To check whether the texture features obtained when the radius is 3 are indeed more effective at improving the image recognition accuracy, we set different radii and conducted experiments on self-made data sets with MDAM-DRNet. These experimental results are shown in Table 4.

**Table 4:** Experimental results with different radius.

**Radius**	**Accuracy**	**Precision_**macro**_**	**Recall_**macro**_**	**F1_**macro**_**
	**(%)**	**(%)**	**(%)**	**(%)**
1	90.31	90.34	90.32	90.33
2	93.92	93.95	93.94	93.95
3	95.79	95.76	95.79	95.77
4	94.52	94.56	94.53	94.55
5	91.51	91.54	91.52	91.53

We see that when the domain radius is set to 3, the recognition accuracy of MDAM-DRNet is 95.79%, and the F1 score is 95.77%, each exceeding that when the radius is set to other values. Therefore, the texture features obtained when the radius is 3 are optimal for enabling the network to extract the key information for strawberry leaf diseases' classification.

#### Test Experiment for the Optimal Value of α

**Figure 9:**
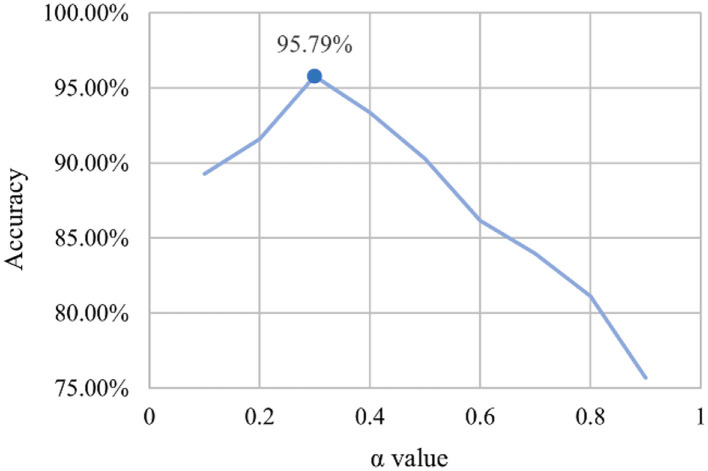
Corresponding relationship between α value and accuracy.

In equation 4, the α value is multiplied by the minimum characteristic to compensate. To determine the appropriate α value, we set different α values and applied MDAM-DRNet to the strawberry leaf data set we collected. This experiment's results are shown in Figure 9.

Evidently, the greatest recognition accuracy was obtained when the x value is 0.3. If the value of α is too small, attention is focused on the global features, and the smaller features go ignored. But if the value of α is too large, attention is shifted to focus on the smaller features, while ignoring the effective features. Both cases will impact the extraction of important feature information by MDAM.

#### Experiment for the Recognition Effect of MDAM-DRNet on Early Disease Images of Strawberry Leaves

In order to test the recognition effect of the model on the early disease of strawberry leaves, we screened the images in the self-made data set. A total of 3,768 images of early strawberry leaf diseases were obtained, these were then divided into a training set, test set, and verification set according to a 3:1:1 ratio. There were 2,260 images in the training set and 754 images in each test set and verification set. We tested the recognition effect of ResNeXt and this paper's proposed MDAM-DRNet model for the early incidence of six disease types of strawberry leaves in the data set. According to the experimental results in Table 5, the recognition accuracy for early diseases of strawberry leaves is significantly improved when using MDAM-DRNet compared with ReNeXt, by about 9.16%. The recognition accuracy of both models was lower for white spot and brown spot because these two diseases cause dark, small round spots in their early stage, whose color and texture are difficult to distinguish. However, the recognition accuracy of MDAM-DRNet for these two kinds of diseases was still >90%, indicating our proposed model proposed is well able to distinguish similar features. In addition, MDAM-DRNe had a high recall and F1 scores for each category, indicating this new method is adept at recognizing leaf diseases in their early stage of development.

**Table 5:** Effect of MDAM-DRNet model on early disease identification of strawberry leaves.

**Methods**	**Categories**	**Number of pictures tested**	**Precision (%)**	**Recall (%)**	**F1 (%)**	**Accuracy (%)**
ResNeXt	Powdery mildew	645	83.72	80.60	82.13	83.00
	Leaf spot	597	79.85	82.40	81.10	
	Botrytis cinerea	642	81.25	83.87	82.54	
	Anthracnose	622	83.06	79.85	81.42	
	Verticillium wilt	573	82.61	84.82	83.70	
	Leaf scorch	689	81.16	80.58	80.87	
MDAM-DRNet	Powdery mildew	645	93.02	90.91	91.95	92.16
	Leaf spot	597	90.76	92.31	91.53	
	Botrytis cinerea	642	92.97	93.70	93.33	
	Anthracnose	622	93.55	92.80	93.17	
	Verticillium wilt	573	92.17	91.38	91.77	
	Leaf scorch	689	90.58	91.91	91.24	

#### The Comparative Experiment of MDAM-DRNet and ResNeXt

We next conducted a comparative experiment between MDAM-DRNet and ResNeXt, using the self-made data set, to verify the optimization of MDAM-DRNet relative to ResNeXt. This paper verifies the optimization effect of the network by comparing the evaluation indicator values obtained for MDAM-DRNet and ResNeXt applied to the same strawberry leaf disease data set.

**Figure 10:**
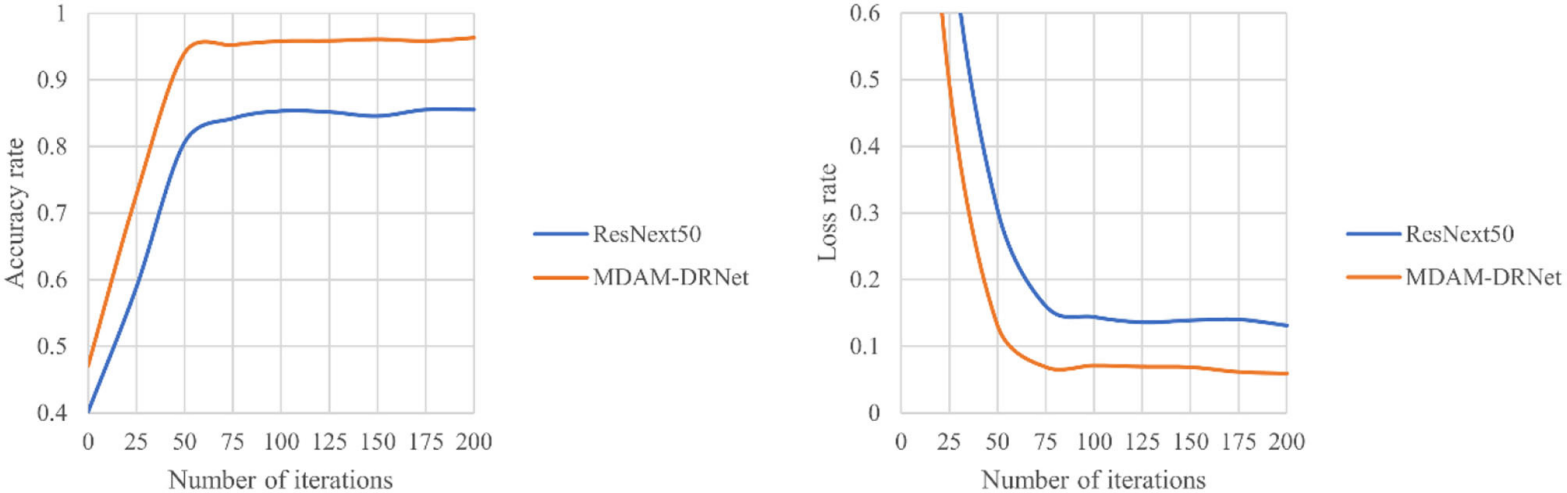
The identification accuracy curves of MDAM-DRNet and ResNeXt for strawberry leaf diseases under the same data set.

According to the experimental results in Table 6, the recognition accuracy of MDAM-DRNet for early-stage diseases of strawberry leaves is significantly improved over ResNeXt, by about 9.67%. The recognition accuracy of MDAM-DRNet for healthy leaves, powdery mildew, leaf spot, *Botrytis cinerea*, anthracnose, verticillium wilt, and leaf scorch was > 95%, and the recognition accuracy of leaf spot and *Botrytis cinerea* was relatively low. This is because the early symptoms of the white spot are not readily apparent, allowing it to be mistaken for another kind of disease, and the early disease images of gray mold accounted for a high proportion, along with texture features that are relatively complex. The recognition accuracy of MDAM-DRNet for each category is at least 93%, and its recall is above 95%, with an F1 score higher than 94%, thus indicating the network has a good classification effect for strawberry leaf diseases. Figure 10 shows the loss and accuracy curves obtained when the MDAM-DRNet and ResNeXt were trained on the same date set, for six strawberry leaf disease images and one healthy strawberry leaf image.

**Table 6:** Recognition results of ResNeXt and MDAM-DRNet for strawberry leaf diseases.

**Methods**	**Categories**	**Precision (%)**	**Recall (%)**	**F1 (%)**	**Accuracy**
ResNeXt	Healthy leaves	89.19	87.33	88.25	86.12
	Powdery mildew	86.23	86.75	86.49	
	Leaf spot	84.11	85.33	84.72	
	Botrytis cinerea	84.06	84.94	84.50	
	Anthracnose	86.52	87.38	86.95	
	Verticillium wilt	86.03	85.69	85.86	
	Leaf scorch	86.49	85.29	85.89	
MDAM-DRNet	Healthy leaves	98.07	95.31	96.97	95.79
	Powdery mildew	96.01	96.78	96.39	
	Leaf spot	94.30	95.07	94.68	
	Botrytis cinerea	93.58	95.97	94.76	
	Anthracnose	95.90	97.04	96.46	
	Verticillium wilt	95.69	95.49	95.59	
	Leaf scorch	96.77	94.86	95.81	

**Figure 11:**
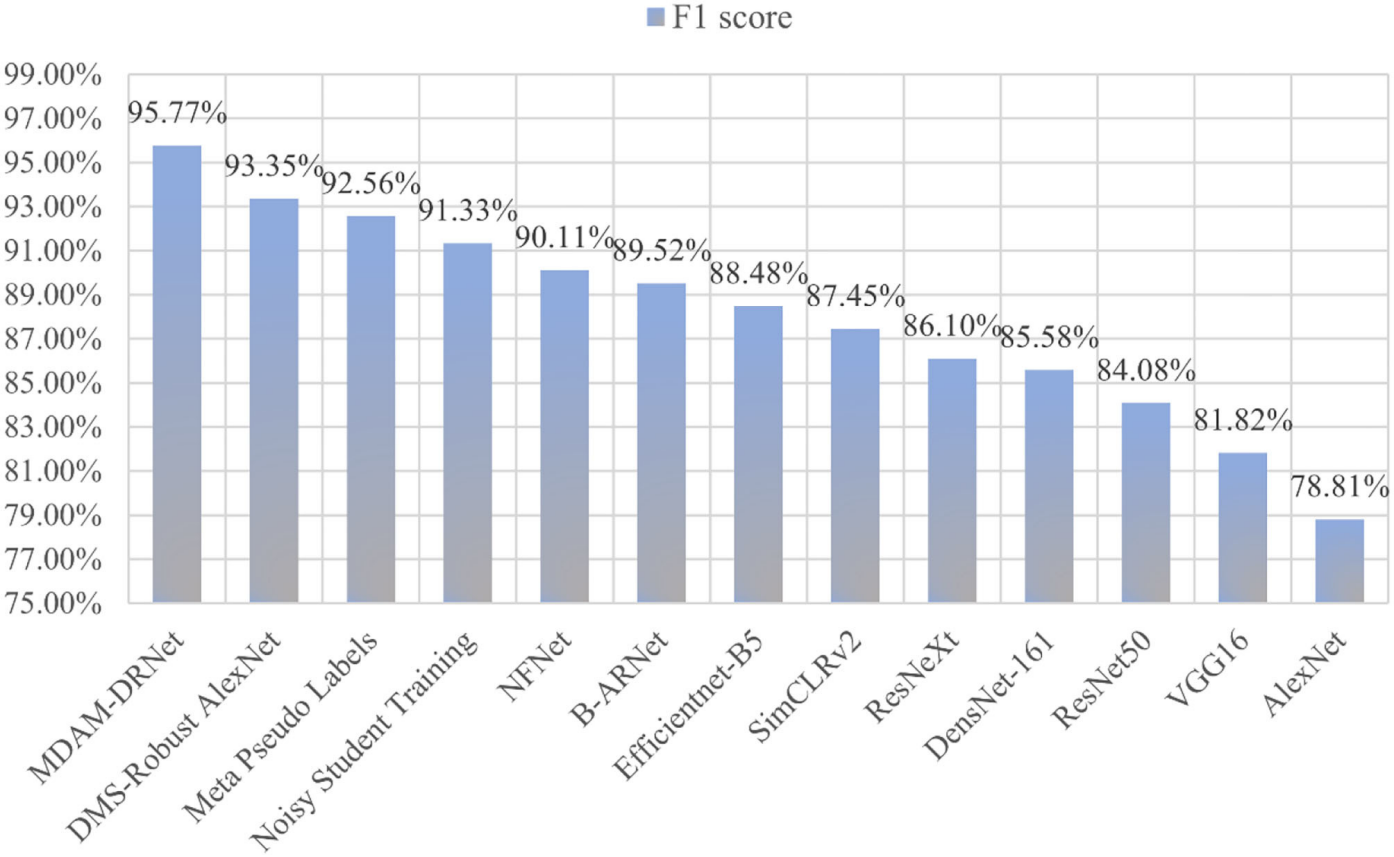
F1 score histogram of each method.

The experimental results in Figure 11 show that when the epoch number of the MDAM-DRNet network reaches 75, the accuracy curve converges and flattens, and its highest recognition accuracy exceeds 95%. When the number of iterations of the ResNeXt network reaches 50, the accuracy curve converges and flattens, and its highest recognition accuracy is more than 85%, which this lower than that of the MDAM-DRNet. The convergence speed of MDAM-DRNet was slightly slower than that of ResNeXt, but it significantly improved the accuracy of strawberry leaf disease identification.

#### Ablation Experiment

This was done to verify the effect of incorporating the MDAM attention mechanism and dual-channel structure of ACRI-LBP and the color correlogram into the ResNeXt model on the image recognition accuracy of strawberry leaf diseases. Through ablation experiments, we compared the recognition ability of strawberry leaf disease images of the following five models and conducted experiments on the same data set under the same experimental environment.

According to their results in Table 7, ResNeXt's strawberry leaf disease recognition accuracy is the lowest among the five models, and the single-use of color or texture features for classification tasks did little to improve accuracy. But after adding MDAM or dual-channel structure to the ResNeXt model, although the number of parameters and training time both increased, overall accuracy is greatly improved. The final superposition effect is more than adequate, having a recognition accuracy of at least 95%, with an F1 score of 95.77%. This proves the modified model is effective for the identification of various diseases afflicting strawberry plants.

**Table 7:** Each model corresponds to the recognition accuracy of each strawberry leaf image.

**Network**	**Accuracy (%)**	**Precision_**macro**_ (%)**	**Recall_**macro**_ (%)**	**F1_**macro**_ (%)**	**Parameters**	**Training time**
ResNeXt	84.09	84.06	84.09	84.08	25M	9 h 8 min
ResNeXt+color correlogram	84.58	84.58	84.57	84.57	25M	9 h 49 min
ResNeXt+ACRI-LBP	83.52	83.51	83.48	83.50	25M	10 h 20 min
MDAM-RNet	90.22	90.19	90.22	90.21	28M	9 h 39 min
DRNet	88.16	88.12	88.14	88.13	27M	11 h 36 min
MDAM-DRNet	95.79	95.76	95.79	95.77	30M	12 h 10 min

#### An Experiment Comparing the Recognition Rate With Other Networks

To verify the performance of the MDAM-DRNet model in the current network, the classification performance of the MDAM-DRNet network model was tested vis-à-vis an existing partial supervised model and a semi-supervised model. Among the models selected for this experiment, AlexNet (Krizhevsky et al., 2012), VGG16 (Simonyan and Zisserman, 2014), Efficientnet-B5 (Tan and Le, 2019), and ResNet50, ResNeXt, and DensNet-161 (Huang et al., 2017) are the most widely used supervision models at present. Noisy Student Training (Xie et al., 2020a), Meta Pseudo Labels (Pham et al., 2021), and SimCLRv2 (Chen T. et al., 2020) are advanced semi-supervised models developed in the past two years; likewise, the B-ARNet model, DMS-Robust Alexnet model, and NFNet (Brock et al., 2021) model are advanced supervision models proposed in the last two years. The respective recognition accuracy of the above 13 models for seven kinds of strawberry leaf images (6 strawberry leaf diseases and a control image [healthy strawberry leaf] in the same strawberry leaf disease data set is conveyed in Table 8.

**Table 8:** The recognition accuracy of 13 models for 7 categories of the same strawberry leaf disease data set.

**Network**	**Healthy**	**Powdery**	**Leaf**	***Botrytis***	**Anthracnose**	**Verticillium**	**Leaf**
	**leaves (%)**	**mildew (%)**	**spot (%)**	***cinerea* (%)**	**(%)**	**wilt (%)**	**scorch (%)**
AlexNet	81.85	76.65	80.86	79.92	77.93	76.39	78.02
VGG16	83.40	81.04	82.08	81.99	82.27	81.11	80.85
ResNet50	86.87	83.83	81.87	81.78	84.18	83.78	86.09
ResNeXt	89.19	86.23	84.11	84.06	86.52	86.04	86.49
DensNet-161	88.80	85.63	83.71	83.64	85.94	85.63	85.69
Efficientnet-B5	92.28	87.62	87.78	86.96	88.87	87.68	88.10
Noisy Student Training	94.40	91.02	89.82	91.10	91.21	91.38	90.32
Meta Pseudo Labels	95.37	92.42	92.46	91.72	92.58	91.99	91.33
SimCLRv2	91.12	88.62	86.35	85.51	87.70	86.45	86.29
B-ARNet	92.66	89.62	87.37	87.99	90.63	89.53	88.71
DMS-Robust Alexnet	95.75	92.81	93.08	92.34	92.38	93.22	93.75
*NFNet*	93.24	90.42	87.78	87.78	90.23	89.12	92.14
*MDAM-DRNet*	98.07	96.01	94.30	93.58	95.90	95.69	96.77

Compared with other network models, the MDAM-DRNet model proposed in this paper has higher recognition accuracy for the six diseases, being higher than 93%. Thus, the application value of the MDAM-DRNet model for strawberry leaf disease detection is confirmed. To further evaluate the performance of this model, accuracy, precision, recall, and F1 scores as evaluation indicators were also compared among models: these results are in Table 9.

**Table 9:** Test results of 13 models on the same strawberry leaf disease data set.

**Network**	**Accuracy (%)**	**Precision_**macro**_ (%)**	**Recall_**macro**_ (%)**	**F1_**macro**_ (%)**
AlexNet	78.81	78.80	78.81	78.81
VGG16	81.82	81.81	81.83	81.82
ResNet50	84.09	84.06	84.09	84.08
ResNeXt	86.12	86.09	86.10	86.10
DensNet-161	85.61	85.58	85.58	85.58
Efficientnet-B5	88.50	88.47	88.49	88.48
Noisy Student Training	91.34	91.32	91.34	91.33
Meta Pseudo Labels	92.57	92.55	92.56	92.56
SimCLRv2	87.47	87.43	87.46	87.45
B-ARNet	89.54	89.50	89.54	89.52
DMS-Robust Alexnet	93.35	93.99	93.36	93.35
NFNet	90.14	90.10	90.12	90.11
MDAM-DRNet	95.79	95.76	95.79	95.77

We can see that the accuracy, precision, recall, and F1 score of our proposed MDAM-DRNet proposed, respectively, were 95.79, 95.76, 95.79, and 95.77%, exceeding those of other models. This substantiates the MDAM-DRNet model's excellent recognition ability for strawberry leaf diseases. To more intuitively compare model accuracy, histograms were drawn (Figure 11); from these, one can clearly see that the MDAM-DRNet has outstanding recognition accuracy.

**Figure 12:**
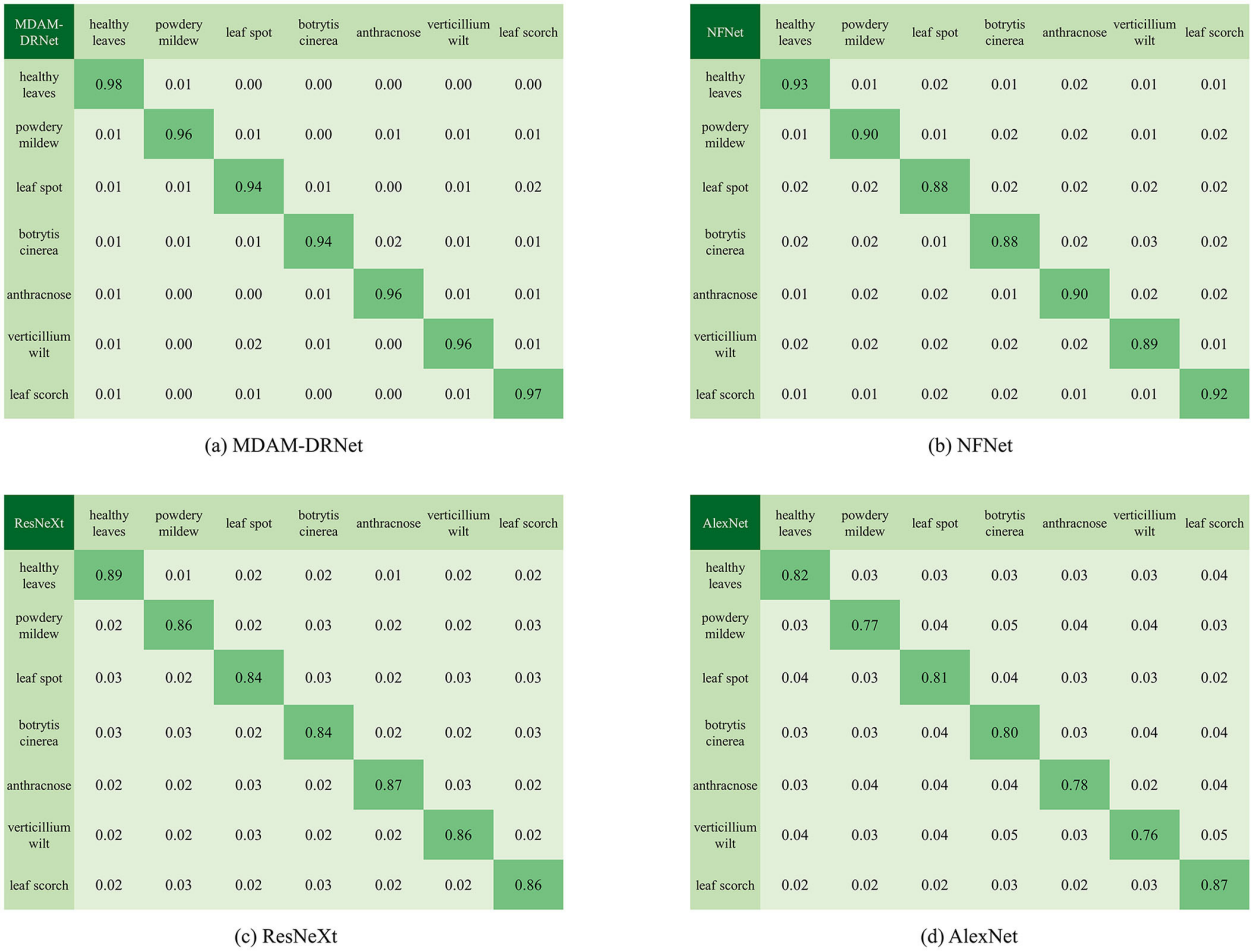
Confusion matrix corresponding to MDAM-DRNet, B-ARNet, ResNeXt, and CNN models.

Among the evaluation indicators of machine learning, in addition to those listed in Table 9, there is also a confusion matrix (also known as a possibility table or error matrix). It is a specific matrix used to visualize the performance of an algorithm, usually one under supervised learning (for unsupervised learning, it is usually called a matching matrix). Each column represents the predicted value and each row represents the actual category. This is very important because, in the actual classification, TP and FP values are the most direct indicators that ultimately determine whether the classification is indeed correct, and the F1 value comprehensively embodies these two critical indicators. As Figure 12 shows, we calculated the confusion matrix based on the experimental results of the MDAM-DRNet, NFNet, ResNeXt, and AlexNet models.

In this confusion matrix, the values on the diagonal are all the correct prediction results, and the remaining values are the wrong prediction results arising from the model's misjudgment. Each row of the matrix represents the real category, and each column of the matrix represents the prediction label of the model. Evidently, the MDAM-DRNet proposed in this paper has a robust classification effect for strawberry leaf diseases: compared with NFNet, ResNeXt, and AlexNet, the number of successful predictions on the diagonal is higher than that attained by other models. Importantly, its performance excelled at detecting/identifying leaf spot diseases with small, cryptic symptoms. Notably, the network framework of MDAM-DRNet was able to correctly classify (more than 94% of cases) two easily confused diseases, leaf spot, and anthracnose. This is because, in its algorithm, the comprehensive weight obtained by MDAM from weights in different directions is soundly aggregated, enabling it to learn the contextual relationship of strawberry leaf diseases, mitigating their similarity to enhance their classification accuracy.

## Discussion

In order to better verify the generalization ability of our MDAM-DRNet model, this paper conducted supplementary experiments on three open data sets of leaf diseases: PlantVillage (Rauf et al., 2019), Citrus (Singh et al., 2020), and PlantDoc (Tan and Le, 2019). Among them, PlantVillage is a multi-category laboratory data set, citrus is a laboratory data set with a small number of categories, and PlantDoc is a multi-category non-laboratory dataset. As neither PlantVillage nor Citrus is already divided into a training set and test set, we divided their data sets into two parts using this training set: a test set ratio of 8:2. The categories of these three public data sets, their number of training set images, and their number of test set images are in Table 10.

**Table 10:** Category of three public data sets, number of training set pictures, and number of test set pictures.

**Dataset**	**Category**	**Training**	**Testing**
PlantVillage	38	43,447	10,862
Citrus	5	487	122
PlantDoc	27	2,334	236

A total of 12 models—AlexNet, VGG16, ResNet50, ResNeXt, DensNet-161, Efficientnet-B5, Noisy Student Training, Meta Pseudo Labels, SimCLRv2, B-ARNet, DMS-Robust Alexnet, and NFNet—were selected and tested against the three public data sets. The experimental results are presented in Table 11. According to these, the recognition accuracy of our proposed network on PlantVillage, Citrus, and PlantDoc data sets is 98.04, 98.36, and 90.16% respectively. The test of MDAM-DRNet using the laboratory data sets revealed a good recognition effect, which is equivalent to the recognition accuracy of an advanced network. The recognition accuracy on the non-laboratory data set (PlantDoc) was >90%, exceeding that of the other model networks, indicating that this paper's proposed network is applicable to real-world environments.

**Table 11:** Recognition accuracy of 13 models tested on three public data sets.

**Network**	**PlantVillage**	**Citrus**	**PlantDoc**
	**Classification accuracy (%)**	**Classification accuracy (%)**	**Classification accuracy (%)**
AlexNet	96.11	97.46	68.85
VGG16	94.53	96.61	65.57
ResNet50	97.66	98.31	79.51
ResNeXt	98.49	98.73	82.79
DensNet-161	95.27	97.46	72.13
Efficientnet-B5	98.40	99.15	83.61
Noisy Student Training	91.85	93.22	82.79
Meta Pseudo Labels	93.11	94.49	85.25
SimCLRv2	90.59	92.37	84.43
B-ARNet	99.03	99.15	87.70
DMS-Robust Alexnet	98.18	98.73	86.89
NFNet	99.34	99.58	89.34
MDAM-DRNet	98.26	99.15	90.16

## Conclusion

In tackling the current problem of recognition accuracy of strawberry leaf disease by image recognition models that are not high, leaving it difficult to distinguish the early-stage disease categories, our paper improves the functioning of ResNeXt for this task. The key innovations of the image recognition network MDAM-DRNet designed here for strawberry leaf diseases are as follows:

1. The color feature path is added to obtain the color features in a strawberry leaf disease image. The color feature path combines the color correlogram and ResNeXt structure to analyze the texture features, which effectively reduces the color interference of other objects in the background and greatly reduces the difficulty of recognition in color extraction.
2. The texture feature path is added to obtain the texture features in a strawberry leaf image. The texture feature path combines ACRI-LBP and ResNeXt structure to analyze the texture features, enabling deeper feature extraction, effectively filtering out the interference of non-feature texture information, which greatly reduces the difficulty of recognition in texture extraction.
3. MDAM is introduced into the main frame road path to extract multi-directional attention, which can dynamically weigh the characteristic data of the region of interest from different directions. This improves the attention of the network to the key region and overcomes the identification difficulty caused by the target's small size. Meanwhile, in the main frame, the ELU function is applied to improve the anti-interference ability of the network.

Compared with the traditional ResNeXt model, the newly designed MDAM-DRNet network in this paper strengthens the recognition accuracy of strawberry leaf diseases, and our model's effectiveness is corroborated by a suite of experiments. In this paper, images of strawberry leave in different periods and regions were collected in representative strawberry planting areas in southern China. Through deep learning and comparison of different models, strawberry leaf diseases in their natural environmental settings are identified and detected, for which high accuracy is achievable. Hence, the MDAM-DRNet network in this paper can aid fruit farmers in accurately monitoring the disease situation of leaves in strawberry orchards, for timely control of disease according to its type, by curtailing its spread. In follow-up work, the new model will be tested in real-world agricultural situations, to contribute to the economic production of strawberries and realize its potentially broader benefits for society as soon as possible.

## Data Availability Statement

The raw data supporting the conclusions of this article will be made available by the authors, without undue reservation.

## Author Contributions

TL: methodology, writing—original draft preparation, and conceptualization. RY: software, data acquisition, and formal analysis. WZ: model guidance and resources. MH: validation, project administration, funding acquisition, and supervision. LL: visualization and writing—review and editing. All authors have read and agreed to the published version of the manuscript.

## Funding

This work was supported by Changsha Municipal Natural Science Foundation (Grant No. kq2014160); in part by the Natural Science Foundation of Hunan Province (Grant No. 2021JJ41087); in part by Hunan Key Laboratory of Intelligent Logistics Technology (Grant No. 2019TP1015).

## Conflict of Interest

The authors declare that the research was conducted in the absence of any commercial or financial relationships that could be construed as a potential conflict of interest.

## Publisher's Note

All claims expressed in this article are solely those of the authors and do not necessarily represent those of their affiliated organizations, or those of the publisher, the editors and the reviewers. Any product that may be evaluated in this article, or claim that may be made by its manufacturer, is not guaranteed or endorsed by the publisher.

## References

[B1] Brock A. De S. Smith S. L. Simonyan K. (2021). “High-performance large-scale image recognition without normalization”, in International Conference on Machine Learning: PMLR PMLR (New York, NY: PMLR), 1059–1071.

[B2] Chakraborty S. Paul S. Rahat-uz-Zaman M. (2021). ”Prediction of Apple Leaf Diseases Using Multiclass Support Vector Machine”, in 2021 2nd International Conference on Robotics, Electrical and Signal Processing Techniques (ICREST) (Dhaka: IEEE), 147–151.

[B3] Chen X. Zhou G. Chen A. Yi J. Zhang W. Hu Y.. (2020). Identification of tomato leaf diseases based on combination of ABCK-BWTR and B-ARNet. Comput. Electron. Agricult. 178, 105730. 10.1016/j.compag.2020.105730

[B4] Chen T. Kornblith S. Swersky K. Norouzi M. Hinton G. (2020). Big self-supervised models are strong semi-supervised learners. Adv. Neural Inf. Process. Syst. 33, 22243–22255. 10.48550/ARXIV.2006.10029

[B5] Clevert D.-A. Unterthiner T. Hochreiter S. (2015). Fast and Accurate Deep Network Learning by Exponential Linear Units (ELUs). arXiv [Preprint]. arXiv:1511.07289.

[B6] Cohn T. Hoang C. D. V. Vymolova E. Yao K. Dyer C. Haffari G.. (2016). Incorporating Structural Alignment Biases into an Attentional Neural Translation Model. Lisbon, Portugal: Association for Computational Linguistics, 876–885.

[B7] Dhaka V. S. Meena S. V. Rani G. Sinwar D. Kavita Ijaz M.F.. (2021). A Survey of Deep Convolutional Neural Networks Applied for Prediction of Plant Leaf Diseases. Sensors 21, 4749. 10.3390/s21144749 34300489 PMC8309553

[B8] Fekriershad S. Tajeripour F. (2017). Color texture classification based on proposed impulse-noise resistant color local binary patterns and significant points selection algorithm. Sens. Rev. 37, 33–42. 10.1108/SR-07-2016-0120

[B9] Guo Z. Zhang L. Zhang D. (2010). A completed modeling of local binary pattern operator for texture classification. IEEE Trans. Image Process. 19, 1657–1663. 10.1109/TIP.2010.2044957 20215079

[B10] He K. Zhang X. Ren S. Sun J. (2016). “Deep residual learning for image recognition”, in: Proceedings of the IEEE conference on computer vision and pattern recognition (Las Vegas, NV: IEEE), 770-778. 10.1109/CVPR.2016.90

[B11] Huang G. Liu Z. Maaten L. V. D. Weinberger K. Q. (2017). “Densely connected convolutional networks”, in 2017 IEEE Conference on Computer Vision and Pattern Recognition (CVPR) (Honolulu, HI: IEEE), 2261–2269.

[B12] Huang S. Zhou G. He M. Chen A. Zhang W. Hu Y.. (2020). Detection of peach disease image based on asymptotic non-local means and PCNN-IPELM. IEEE Access. 8, 136421–136433. 10.1109/ACCESS.2020.3011685

[B13] Jing H. Kumar S. R. Mitra M. Wei-Jing Z. Zabih R. (1997). ”Image indexing using color correlograms”, in Proceedings of IEEE Computer Society Conference on Computer Vision and Pattern Recognition (San Juan: IEEE), 762–768.

[B14] Kavitha J. C. Suruliandi A. (2016). “Texture and color feature extraction for classification of melanoma using SVM”, in 2016 International Conference on Computing Technologies and Intelligent Data Engineering (ICCTIDE'16) (Kovilpatti: IEEE), 1–6.

[B15] Kim B. Han Y-. K. Park J-. H. Lee J. (2021). Improved Vision-Based Detection of Strawberry Diseases Using a Deep Neural Network. Front. Plant Sci. 11, 559172. 10.3389/fpls.2020.559172 33584739 PMC7874225

[B16] Kingma D. P. Ba J. (2014). Adam: a method for stochastic optimization. arXiv preprint arXiv:1412, 6980.

[B17] Krizhevsky A. Sutskever I. Hinton G. E. (2012). Imagenet classification with deep convolutional neural networks. Adv. Neural Inf. Process. Syst. 25. 60, 84–90. 10.1145/3065386

[B18] Kundu N. Rani G. Dhaka V. S. Gupta K. Nayak S. C. Verma S.. (2021). IoT and interpretable machine learning based framework for disease prediction in pearl millet. Sensors 21, 5386. 10.3390/s21165386 34450827 PMC8397940

[B19] Kusumandari D. E. Adzkia M. Gultom S. P. Turnip M. Turnip A. (2019). Detection of strawberry plant disease based on leaf spot using color segmentation. J. Phys. Conf. Ser. 1230, 012092. 10.1088/1742-6596/1230/1/012092

[B20] Lei J. J. Jiang S. Ma R. Y. Xue L. Zhao J. Dai H. P.. (2021). ”Current status of strawberry industry in China“: International Society for Horticultural Science (ISHS) (San Juan: ISHS), 349–352.

[B21] Li Y. Chao X. (2021). Semi-supervised few-shot learning approach for plant diseases recognition. Plant Meth. 17, 68. 10.1186/s13007-021-00770-1 34176505 PMC8237441

[B22] Liu X. Chen S. Song L. Wozniak M. Liu S. (2021). Self-attention negative feedback network for real-time image super-resolution. J. King Saud Univ. Comput. Inf. Sci. 10.1016/j.jksuci.2021.07.014

[B23] Luong T. Pham H. Manning C. D. (2015). Effective Approaches to Attention-Based Neural Machine Translation. Lisbon, Portugal: Association for Computational Linguistics, 1412–1421. 10.18653/v1/D15-1166

[B24] Lv M. Zhou G. He M. Chen A. Zhang W. Hu Y.. (2020). Maize leaf disease identification based on feature enhancement and DMS-robust alexnet. IEEE Access. 8, 57952–57966. 10.1109/ACCESS.2020.2982443

[B25] Mäenpää T. Pietikäinen M. (2005). “Texture analysis with local binary patterns”, in Handbook of Pattern Recognition and Computer Vision. Singapore: World Scientific, 197-216.

[B26] Ojala T. Pietikainen M. Maenpaa T. (2002). Multiresolution gray-scale and rotation invariant texture classification with local binary patterns. IEEE Transac. Pattern Anal. Mac. Intell. 24, 971–987. 10.1109/TPAMI.2002.1017623

[B27] Opitz J. Burst S. (2019). Macro F1 and Macro F1. arXiv [Preprint]. arXiv:1911.03347.

[B28] Pham H. Dai Z. Xie Q. Le Q. V. (2021). ”Meta pseudo labels”, in 2021 IEEE/CVF Conference on Computer Vision and Pattern Recognition (CVPR) (Nashville, TN: IEEE), 11552–11563.

[B29] Rauf H. T. Saleem B. A. Lali M. I. U. Khan M. A. Sharif M. Bukhari S. A. C.. (2019). A citrus fruits and leaves dataset for detection and classification of citrus diseases through machine learning. Data Brief. 26, 104340. 10.1016/j.dib.2019.104340 31516936 PMC6731382

[B30] Simonyan K. Zisserman A. (2014). Very deep convolutional networks for large-scale image recognition. arXiv preprint arXiv:1409, 1556.

[B31] Singh D. Jain N. Jain P. Kayal P. Kumawat S. Batra N.. (2020). Plantdoc: a dataset for visual plant disease detection, in Proceedings of the 7th ACM IKDD CoDS and 25th COMAD (New York, NY: Association for Computing Machinery), 249–253.

[B32] Skrovankova S. Sumczynski D. Mlcek J. Jurikova T. Sochor J. (2015). Bioactive Compounds and Antioxidant Activity in Different Types of Berries. Int. J. Mol. Sci. 16, 24673–706. 10.3390/ijms161024673 26501271 PMC4632771

[B33] Tan M. Le Q. (2019). “Efficientnet: rethinking model scaling for convolutional neural networks”, in International conference on machine learning: PMLR (New York, NY: PMLR), 6105-6114.

[B34] Tu Z. Lu Z. Liu Y. Liu X. Li H. (2016). Modeling Coverage for Neural Machine Translation. *arXiv preprint arXiv:1601.04811*.

[B35] Velickovic P. Cucurull G. Casanova A. Romero A. Lio P. Bengio Y.. (2017). Graph attention networks. Stat. 1050, 20. 10.48550/ARXIV.1710.10903

[B36] Wang X. Girshick R. Gupta A. He K. (2018). ”Non-local neural networks”, in 2018 IEEE/CVF Conference on Computer Vision and Pattern Recognition (Honolulu, HI: IEEE), 7794–7803.

[B37] Wang Z. Cang T. Qi P. Zhao X. Xu H. Wang X.. (2015). Dissipation of four fungicides on greenhouse strawberries and an assessment of their risks. Food Control 55, 215–220. 10.1016/j.foodcont.2015.02.050

[B38] Wei-Ying M. Hong Jiang Z. (1998). “Benchmarking of image features for content-based retrieval”, in Conference Record of Thirty-Second Asilomar Conference on Signals, Systems and Computers (Cat. No.98CH3628), vol. 251, 253–257.

[B39] Xiao J-. R. Chung P-. C. Wu H-. Y. Phan Q-. H. Yeh J-. L. A. Hou M. T.. (2021). Detection of strawberry diseases using a convolutional neural network. Plants. 10, 31. 10.3390/plants10010031 33375537 PMC7823414

[B40] Xie Q. Luong M.-T. Hovy E. Le Q.V. (2020a). “Self-training with noisy student improves imagenet classification”, in: Proceedings of the IEEE/CVF conference on computer vision and pattern recognition (Seattle, WA: IEEE), 10687–10698. 10.1109/CVPR42600.2020.01070

[B41] Xie S. Girshick R. Dollár P. Tu Z. He K. (2017). ”Aggregated residual transformations for deep neural networks”, in Proceedings of the IEEE conference on computer vision and pattern recognition (Honolulu, HI: IEEE), 1492–1500.

[B42] Xie X. Ma Y. Liu B. He J. Li S. Wang H.. (2020b). A Deep-Learning-Based Real-Time Detector for Grape Leaf Diseases Using Improved Convolutional Neural Networks. Front. Plant Sci. 11, 751. 10.3389/fpls.2020.00751 32582266 PMC7285655

[B43] Yang G.-f. Yang Y. He Z.-k. Zhang X.-y. He Y. (2022). A rapid, low-cost deep learning system to classify strawberry disease based on cloud service. J. Integr. Agricult. 21, 460–473. 10.1016/S2095-3119(21)63604-3

[B44] Zhang W. Hu J. Zhou G. He M. (2020). Detection of Apple Defects Based on the FCM-NPGA and a Multivariate Image Analysis. IEEE Access. 8, 38833–38845. 10.1109/ACCESS.2020.2974262

